# Supplementary material for: Comparison of diagnostic algorithms used in guidelines on nutritional anaemias in adults
Source: PLoS One. 2025 Oct 3;20(10):e0333688. doi: 10.1371/journal.pone.0333688 (PMC12494270; doi:10.1371/journal.pone.0333688)
Supplement: Attachment — Submitted filename: PONE-D-25-00163 (2)PLOSOneAnaemia_JJO.pdf [file pone.0333688.s004.pdf]

PLOS ONE

Diagnostic algorithms in guidelines on anaemia: a systematic review

--Manuscript Draft--

**Manuscript Number:**	PONE-D-25-00163
**Article Type:**	Research Article
**Full Title:**	Diagnostic algorithms in guidelines on anaemia: a systematic review
**Short Title:**	Diagnostic algorithms in guidelines on anaemia
**Corresponding Author:**	Anne Margreet De Jong, PhD<br>Atalmedial diagnostic centre<br>Amsterdam, NETHERLANDS, KINGDOM OF THE
**Keywords:**	general practice, anaemia, iron deficiency, ferritin, vitamin B12 deficiency, folate deficiency, guidelines, laboratory aspects
**Abstract:**	Background<br>Anaemia is highly prevalent and is commonly caused by nutritional deficiencies. Guidelines often include an algorithm to find this underlying cause. Here, we compared diagnostic algorithms and suggested laboratory tests for anaemia in clinical practice guidelines in countries with similar healthcare systems, focusing on iron, vitamin B12 and folate deficiency anaemia in the general adult population.<br><br>Methods<br>We searched for guidelines on anaemia in the Trip, Guidelines International Network and country specific databases. To be selected for this systematic review, the guidelines had to include diagnostic criteria or algorithms to determine the cause of anaemia in the general adult population.<br><br>Results<br>In total, 14 records were included. For iron deficiency anaemia, guidelines varied in diagnostic criteria ranging from only ferritin up to ferritin in various combinations with a variety of other parameters, with different cut-off values. For vitamin B12 or folate deficiency, besides measurement of vitamin B12 or folate, some guidelines mentioned methylmalonic acid or homocysteine. Quality of evidence underlying cut-offs and parameters was variable, and laboratory aspects were underrepresented.<br><br>Conclusions<br>There was a lot of variation in the included diagnostic algorithms, especially for iron deficiency anaemia. Differences in cut-off values were seen, even when using similar diagnostic strategies. Furthermore, supporting evidence was variable. Further research is needed to determine optimal algorithms. Our results highlight the need for inclusion of relevant laboratory aspects in guidelines, considering appropriate diagnostics and clinical decision limits.
**Order of Authors:**	Margaretha G.M. Roemer, PhD
	Wendy P.J. den Elzen, PhD
	Rosalinde K.E. Poortvliet, PhD
	Jacobijn Gussekloo, Prof. Dr.
	Steef Kurstjens, PhD
	Bauke A. de Boer, PhD
	Anne Margreet De Jong, PhD
**Additional Information:**	
**Question**	**Response**
**Financial Disclosure**	The author(s) received no specific funding for this work.

Powered by Editorial Manager® and ProduXion Manager® from Aries Systems Corporation

Enter a financial disclosure statement that describes the sources of funding for the work included in this submission. Review the submission guidelines for detailed requirements. View published research articles from *PLOS ONE* for specific examples.<br><br>This statement is required for submission and **will appear in the published article** if the submission is accepted. Please make sure it is accurate.<br><br>**Funded studies**<br>Enter a statement with the following details:<br>• Initials of the authors who received each award<br>• Grant numbers awarded to each author<br>• The full name of each funder<br>• URL of each funder website<br>• Did the sponsors or funders play any role in the study design, data collection and analysis, decision to publish, or preparation of the manuscript?<br><br>Did you receive funding for this work?	
**Competing Interests**<br><br>Use the instructions below to enter a competing interest statement for this submission. On behalf of all authors, disclose any competing interests that could be perceived to bias this work—acknowledging all financial support and any other relevant financial or non-financial competing interests.<br><br>This statement is required for submission and **will appear in the published article** if the submission is accepted. Please make sure it is accurate and that any funding sources listed in your Funding Information later in the submission form are also declared in your Financial Disclosure statement.<br><br>View published research articles from	The authors have declared that no competing interests exist.

PLOS ONE for specific examples.<br><br>**NO authors have competing interests**<br><br>Enter: *The authors have declared that no competing interests exist.*<br><br>**Authors with competing interests**<br><br>Enter competing interest details beginning with this statement:<br><br>*I have read the journal's policy and the authors of this manuscript have the following competing interests: [insert competing interests here]*<br><br>* typeset	
**Ethics Statement**<br><br>Enter an ethics statement for this submission. This statement is required if the study involved:<br><br>• Human participants<br>• Human specimens or tissue<br>• Vertebrate animals or cephalopods<br>• Vertebrate embryos or tissues<br>• Field research<br><br>Write "N/A" if the submission does not require an ethics statement.<br><br>General guidance is provided below. Consult the submission guidelines for detailed instructions. **Make sure that all information entered here is included in the Methods section of the manuscript.**	n/a

Powered by Editorial Manager® and ProduXion Manager® from Aries Systems Corporation

**Format for specific study types**<br><br>**Human Subject Research (involving human participants and/or tissue)**<br>• Give the name of the institutional review board or ethics committee that approved the study<br>• Include the approval number and/or a statement indicating approval of this research<br>• Indicate the form of consent obtained (written/oral) or the reason that consent was not obtained (e.g. the data were analyzed anonymously)<br><br>**Animal Research (involving vertebrate animals, embryos or tissues)**<br>• Provide the name of the Institutional Animal Care and Use Committee (IACUC) or other relevant ethics board that reviewed the study protocol, and indicate whether they approved this research or granted a formal waiver of ethical approval<br>• Include an approval number if one was obtained<br>• If the study involved *non-human primates*, add *additional details* about animal welfare and steps taken to ameliorate suffering<br>• If anesthesia, euthanasia, or any kind of animal sacrifice is part of the study, include briefly which substances and/or methods were applied<br><br>**Field Research**<br><br>Include the following details if this study involves the collection of plant, animal, or other materials from a natural setting:<br>• Field permit number<br>• Name of the institution or relevant body that granted permission	
**Data Availability**<br><br>Authors are required to make all data underlying the findings described fully available, without restriction, and from the time of publication. PLOS allows rare exceptions to address legal and ethical concerns. See the PLOS Data Policy and FAQ for detailed information.	Yes - all data are fully available without restriction

Powered by Editorial Manager® and ProduXion Manager® from Aries Systems Corporation

A Data Availability Statement describing where the data can be found is required at submission. Your answers to this question constitute the Data Availability Statement and **will be published in the article**, if accepted.<br><br>**Important:** Stating 'data available on request from the author' is not sufficient. If your data are only available upon request, select 'No' for the first question and explain your exceptional situation in the text box.<br><br>Do the authors confirm that all data underlying the findings described in their manuscript are fully available without restriction?	
**Describe where the data may be found in full sentences. If you are copying our sample text, replace any instances of XXX with the appropriate details.**<br><br>• If the data are **held or will be held in a public repository**, include URLs, accession numbers or DOIs. If this information will only be available after acceptance, indicate this by ticking the box below. For example: *All XXX files are available from the XXX database (accession number(s) XXX, XXX.).*<br>• If the data are all contained **within the manuscript and/or Supporting Information files**, enter the following: *All relevant data are within the manuscript and its Supporting Information files.*<br>• If neither of these applies but you are able to provide **details of access elsewhere**, with or without limitations, please do so. For example:<br><br>*Data cannot be shared publicly because of [XXX]. Data are available from the XXX Institutional Data Access / Ethics Committee (contact via XXX) for researchers who meet the criteria for access to confidential data.*<br><br>*The data underlying the results presented in the study are available from (include the name of the third party*	All relevant data are within the manuscript and its Supporting Information files.

and contact information or URL).<br>• This text is appropriate if the data are owned by a third party and authors do not have permission to share the data.<br><br>* typeset	
Additional data availability information:	

Powered by Editorial Manager® and ProduXion Manager® from Aries Systems Corporation

Manuscript

Click here to access/download;Manuscript;RdE_Diagnostic algorithms in guidelines on anaemia_update.docx

Diagnostic algorithms in guidelines on anaemia: a systematic review

Margaretha G.M Roemer[1,2]*, Wendy P.J. den Elzen[3,4]* , Rosalinde K.E. Poortvliet[5,6], Jacobijn Gussekloo[5,6,7], Steef Kurstjens[8,9], Bauke A. de Boer[1], Anne Margreet de Jong[1]

1. Atalmedial diagnostic centre, Amsterdam, the Netherlands
2. Department of Clinical Chemistry, Haematology & Immunology, Noordwest Ziekenhuisgroep, Alkmaar, the Netherlands
3. Laboratory Specialized Diagnostics & Research, Department of Laboratory Medicine, Amsterdam UMC, University of Amsterdam, Amsterdam, the Netherlands
4. Amsterdam Public Health Research Institute & Amsterdam Gastroenterology and Endocrinology Metabolism, Amsterdam, the Netherlands
5. Department of Public Health and Primary Care, Leiden University Medical Center, Leiden, the Netherlands
6. LUMC Center for Medicine for Older people, Leiden, the Netherlands
7. Department of Internal Medicine, Section of Gerontology and Geriatrics, Leiden University Medical Center, Leiden, the Netherlands
8. Laboratory of Clinical Chemistry and Hematology, Jeroen Bosch Hospital, 's Hertogenbosch, the Netherlands
9. Laboratory of Clinical Chemistry and Laboratory Medicine, Dicoon BV, location Canisius Wilhelmina Hospital, Nijmegen, the Netherlands

* Equal contributions

OrcID

Margaretha G.M Roemer	0000-0003-0220-3496
Wendy P.J. den Elzen	0000-0002-6774-4077
Rosalinde K.E. Poortvliet	0000-0001-6521-8770
Jacobijn Gussekloo	0000-0001-7186-8278
Steef Kurstjens	0009-0008-4409-5079
Bauke A. de Boer	0000-0002-4572-1157
Anne Margreet de Jong	0000-0002-9638-9115

Corresponding author
Dr. Anne Margreet de Jong
Atalmedial Diagnostics Centre, Amsterdam, The Netherlands
Louwesweg 6
P.O. Box 69641
1066 EC Amsterdam
The Netherlands
Email: adejong@atalmedial.nl

Declarations
Conflict of interest: The authors declare no competing interests.
Funding: This study had no external funding source.
Ethical approval: Not applicable.
Author's contributions: Conceptualization, study design and data collection: M.G.M.R, W.P.J.d.E., B.A.d.B. and A.M.d.J.; data preparation and methodology: M.G.M.R, W.P.J.d.E.

and A.M.d.J.; investigation: M.G.M.R, W.P.J.d.E. and A.M.d.J.; writing - original draft preparation: M.G.M.R, W.P.J.d.E. and A.M.d.J; writing - review and editing: M.G.M.R, W.P.J.d.E. and A.M.d.J, R.K.E.P., J.G., S.K., B.A.d.B. All authors have read and agreed to the published version of the manuscript.

Acknowledgements: not applicable.

Number of words: 2499 words

Short title: diagnostic algorithms on anaemia

Keywords: general practice, anaemia, iron deficiency, ferritin, vitamin B12 deficiency, folate deficiency, guidelines, laboratory aspects

Abstract

Background

Anaemia is highly prevalent and is commonly caused by nutritional deficiencies. Guidelines often include an algorithm to find this underlying cause. Here, we compared diagnostic algorithms and suggested laboratory tests for anaemia in clinical practice guidelines in countries with similar healthcare systems, focusing on iron, vitamin B12 and folate deficiency anaemia in the general adult population.

Methods

We searched for guidelines on anaemia in the Trip, Guidelines International Network and country specific databases. To be selected for this systematic review, the guidelines had to include diagnostic criteria or algorithms to determine the cause of anaemia in the general adult population.

Results

In total, 14 records were included. For iron deficiency anaemia, guidelines varied in diagnostic criteria ranging from only ferritin up to ferritin in various combinations with a variety of other parameters, with different cut-off values. For vitamin B12 or folate deficiency, besides measurement of vitamin B12 or folate, some guidelines mentioned methylmalonic acid or homocysteine. Quality of evidence underlying cut-offs and parameters was variable, and laboratory aspects were underrepresented.

Conclusions

There was a lot of variation in the included diagnostic algorithms, especially for iron deficiency anaemia. Differences in cut-off values were seen, even when using similar diagnostic strategies. Furthermore, supporting evidence was variable. Further research is needed to determine optimal algorithms. Our results highlight the need for inclusion of relevant laboratory aspects in guidelines, considering appropriate diagnostics and clinical decision limits.

Introduction

Anaemia is a highly prevalent disorder worldwide affecting 24.8% of the population[1,2]. Anaemia is defined as a low number of red blood cells or a low haemoglobin concentration within the red blood cells[1]. Low haemoglobin concentrations result in a decreased capacity of the blood to carry oxygen to the tissues in the body. Consequently, symptoms include fatigue, weakness, dizziness and shortness of breath. Anaemia is not a diagnosis in itself, but often a presentation of an underlying condition. Anaemia is most frequently caused by nutrient deficiencies due to inadequate diets or inadequate absorption of nutrients, predominantly iron, vitamin B12 and folate. In addition, chronic diseases, infection, kidney failure, and also genetic disorders, bone marrow pathologies, and acute or chronic haemorrhage can cause anaemia[3,4]. It is therefore relevant to diagnose the cause of anaemia to adequately treat the patient.

General practitioners play a crucial role in the diagnosis and management of anaemia. Analysis of anaemia typically involves a complete blood count (CBC) and further investigative assays to pinpoint the specific aetiology. Premature or unnecessary testing can lead to excessive use of diagnostic blood tests, while failure to test anaemic patients can result in incomplete or missed diagnoses or under- of overtreatment. Both scenarios can potentially increase healthcare costs[5-7].

To ensure effective and standardised care, anaemia guidelines have been developed. These guidelines typically encompass initial assessment, diagnostic testing, management and treatment, follow-up and monitoring. Adhering to established clinical guidelines is advised to ensure effective and standardised care. Given the different physiological needs varying by age, sex, ethnicity, pregnancy status or within specific patient groups, a plethora of different guidelines for analysis of anaemia have been developed. Also, different countries have different requirements for the way in which guidelines are developed, who is involved in their development, and what sources are used. Therefore, the aim of this systematic review was to compare the diagnostic algorithms and suggested laboratory tests for anaemia in clinical practice guidelines in countries with similar healthcare systems for the most common causes of anaemia: iron, vitamin B12 and folate deficiency in adults from the primary care population. This may provide guidance for the development of novel guidelines.

Methods

Data sources and searches

First, we searched for guidelines on anaemia in two guideline databases: Trip database (https://www.tripdatabase.com) and Guidelines International Network (G-I-N, http://www.g-i-n.net) on January 12th 2022. These databases were selected to search for evidence-based guidelines across the world. We performed an advanced search for 'anemia' or 'anaemia' in both databases. Second, we performed searches in country specific guideline databases (ao. Germany, UK, USA, Canada, Australia) on October 15th 2023. The most recent version of the guidelines was sought and used on February 29th 2024.

Guideline selection

To be selected for this systematic review, the guidelines had to include diagnostic criteria or algorithms to find the cause of anaemia in the primary care adult population. We focused on nutritional deficiencies (iron, vitamin B12 and folate). Guidelines had to include criteria for adults; specific guidelines for children were excluded. When multiple guidelines from the same society were found, the most recent version was used. Guidelines focusing on specific populations (e.g. anaemia in pregnancy, chronic kidney disease, dialysis patients, heart failure, cancer), guidelines of which full versions were not available in English, Dutch or German and guidelines of which full versions were inaccessible online or withdrawn, were excluded. Two authors (combinations of MR, WdE, AdJ) independently assessed the titles and full texts of the guidelines retrieved during the searches against the inclusion and exclusion criteria, and selected or rejected as appropriate. Disagreement was resolved by consensus.

Data extraction, quality assessment and analysis

The recommendations in the selected guidelines were extracted by two authors (WdE, AdJ) and checked by a third (MR). We rated the quality of the guidelines with a scoring system, based on the evidence underpinning the recommendations. Level 0: no evidence provided, level 1: based on expert opinion, level 2: based on randomised controlled trials / peer reviewed studies before 2010 (for the test of interest), level 3: based on randomised controlled trials / peer reviewed studies 2010 and later (for the test of interest), level 4: based on a systematic review / meta-analysis. Note that guidelines that referred to studies before, as well as after 2010 were evaluated as level 3.

Results

A total of 87 records were identified by the combined searches. Forty-four records were excluded due to the focus on a specific patient group. The remaining 43 full texts were screened and another 29 were excluded in line with the above mentioned exclusion criteria (Figure 1). The 14 included guidelines originated from 6 different countries (US, UK, Germany, Canada, Qatar, the Netherlands).

Patient population

We focused on guidelines in the primary care patient population. Nonetheless, a plethora of different anaemia guidelines were found that included specific organs, patient groups and underlying diseases. Therefore, we decided to select all guidelines focusing on the "general population". This included primary care, as well as secondary care guidelines in otherwise healthy people.

Age definitions for patient populations that were included in the different guidelines were variable. Most of the guidelines did not state a specific age definition[8-15], while others focused on age ≥5 years[16], >12 years[17], ≥16 years[18,19], ≥18 years[20] or on older adults[21]. Although all guidelines acknowledged that older people have a higher risk of developing anaemia, and/or suggested further diagnostics, no age-specific diagnostic algorithms were provided.

Definition of anaemia

According to the World Health Organization (WHO), anaemia is defined as haemoglobin levels <12.0 g/dL in women and <13.0 g/dL in men[1]. Most of the guidelines adhered to this definition[8,9,11-14,17,19,20], although one guideline used <13.5 g/dL in men[16] and one guideline used <11.5 g/dL in women (Table 1)[18]. Three guidelines did not specify a value for low haemoglobin, but referred to local laboratory reference values[10,15,21].

Iron deficiency anaemia

For iron deficiency anaemia, there was a lot of variation in the included diagnostic criteria, as summarised in Table 2. Diagnostic criteria ranged from only ferritin, up to ferritin in various combinations with mean corpuscular volume (MCV), iron, transferrin, transferrin saturation, total iron binding capacity (TIBC), C-reactive protein (CRP) or erythrocyte sedimentation rate (ESR), and reticulocyte haemoglobin content (Ret-Hb) or reticulocytes, with different cut-off values. Of note, even when a similar strategy was used, differences in cut-off values were seen.

Testing of MCV was recommended in seven guidelines, in combination with ferritin[9-12,16,20,21]. Other erythrocyte indices (mean corpuscular haemoglobin, mean corpuscular

haemoglobin concentration, red cell distribution width) were variably mentioned in a minority of the guidelines, and therefore not included in Table 2.

All guidelines included ferritin for the diagnosis of iron deficiency anaemia. Cut-offs ranged from <15 µg/L[15,18], <30 µg/L[8,14,17,20] to <45 µg/L[13] or referred to the local lower limit of reference range[10-12,21]. Two guidelines used different ferritin cut-offs for men and women[9,16].

The fact that ferritin is an acute phase protein and thus increases with infection or inflammation was recognized in all guidelines. Nonetheless, some guidelines only mentioned it[9,12,13,17,21], while other guidelines specified the ferritin level for which iron deficiency could still be considered[11,15,16,18,20] and/or recommended measurement of CRP/ESR to confirm the presence of inflammation[8,10,14]. Additional testing of transferrin saturation when a normal ferritin level was found was recommended in five guidelines[8,10,14,20,21].

Testing of iron itself was recommended in three guidelines, in combination with MCV, ferritin, transferrin, transferrin saturation and/or TIBC[11,12,21]. Lastly, three guidelines recommended examination of Ret-Hb or reticulocytes[8-10].

Vitamin B12 or folate deficiency anaemia

Six vitamin B12 and folate deficiency anaemia guidelines were included. Most of the guidelines contained a combined anaemia diagnostic algorithm with iron deficiency[8,11,12,20,21] and one guideline specifically focused on vitamin B12 and folate deficiency (Table 3)[19]. All guidelines mentioned that vitamin B12 and/or folate anaemia is macrocytic. Four guidelines stated that the anaemia may also be normocytic[8,11,19,21].

For vitamin B12, one guideline recommended a specific cut-off[19], the other guidelines referred to local laboratory reference values. With regards to the use of total vitamin B12 (cobalamin) or active vitamin B12 (holotranscobalamin), four guidelines specifically recommended the (initial) testing of total vitamin B12[8,11,19,20].

In addition to vitamin B12, four guidelines mentioned testing for methylmalonic acid (MMA) and/or homocysteine, always in addition to vitamin B12[12,21] or when the vitamin B12 level is in the lower normal reference range[11,20].

For folate, most guidelines recommended testing for folate only[8,11,21]. Two guidelines included RBC folate as a diagnostic criterion[19,20]. One guideline recommended lactate dehydrogenase (LDH) and reticulocytes for folate, as well as vitamin B12 deficiency[11].

Quality of the evidence

The quality of the included guidelines was evaluated and ranked for the indicated parameters (colour-coded in Table 1, 2 and 3). To define anaemia, WHO haemoglobin cut-offs were used in most guidelines[8,9,11-14,17,19-21]. Given the systematic nature of the evidence and recently unchanged cut-offs[22], this was regarded as level 4. For iron deficiency anaemia, two guidelines

were considered level 4[10,13], five guidelines level 3[8,12,16,17,20], four guidelines level 2[9,11,15,21] and two guidelines level 1[14,18]. It must be noted that guidelines frequently referred to each other, but nonetheless used variable cut-offs. Five out of six vitamin B12 and folate deficiency anaemia guidelines were considered level 3[8,11,12,19,20] and one guideline level 2[21].

Laboratory aspects

Laboratory aspects were only scarcely reported in the included guidelines. Even though laboratory measurements are used in all guidelines, none of the guidelines specified which manufacturer or measurement method was used to determine the specific cut-off values, and the type of material (e.g. plasma or serum) that should be used for the test of interest.

Discussion

In primary care[illegible]BC is one of the most common blood tests, often done as a routine check-up. When a low haemoglobin is found, follow-up tests are used to investigate the cause of anaemia[23]. This systematic review elucidated similarities and differences in diagnostic algorithms in evidence-based guidelines for the most common causes of anaemia: iron, vitamin B12 and folate deficiency. An overview of our observations and recommendations for future guidelines are summarised in Figure 2. In brief, we observed that primary and secondary care guidelines are intertwined, and that there is insufficient attention to [illegible]ersity aspects. Furthermore, a lot of variation was seen in the recommended diagnostic criteria and cut-off values, even in the presence of similar diagnostic strategies. Importantly, even though laboratory testing is proposed in all guidelines, the specifics of these tests are not described.

Patient population and diversity in diagnostic algorithms

Primary care, as well as secondary care guidelines in otherwise healthy people were included. For future guidelines, a focus on either the primary or secondary care setting is endorsed, as well as alignment between the recommendations of primary care and secondary care. Similar misalignments were observed in the field of subclinical hyperthyroidism[24].

Remarkably, no distinctions were made by sex or age in the majority of the diagnostic algorithms. However, research shows that sex differences may be of importance[25]. Nonetheless, the WHO did not find enough evidence for a change of ferritin cut-off values by sex[26]. Additionally, the diagnostic work-up and treatment of anaemia in older patients may be different from other age groups[27-29]. Hence, we recommend including different aspects of diversity, at least age and sex, in the diagnostic algorithms.

Included parameters in diagnostic algorithms

After anaemia is identified, the majority of the guidelines used MCV to define the cause of the anaemia. Nonetheless, a normal MCV does not exclude the presence of iron, vitamin B12 or folate deficiency[30]. Therefore, MCV should be mainly used as a supporting parameter.

For iron deficiency anaemia, ferritin was included in all algorithms. However, the used cut-offs were variable, as has been previously described[31-33], and no proper justification for these cut-offs was provided. Despite an extensive review performed by the WHO, insufficient evidence was found for a consensus on the ferritin threshold and therefore the cut-off of <15 µg/L remained unchanged[26,31,32]. In the presence of inflammation, WHO recommends a cut-off of 70 µg/L to diagnose iron deficiency anaemia[26]. However, most of the guidelines stated a ferritin cut-off of 100 µg/L in this situation. There is no consensus about which additional parameters should be evaluated.

For vitamin B12 and folate deficiency, most guidelines referred to local lower limit of reference values. Given the variation of used cut-offs in literature[34,35], this is a sensible approach. Not all guidelines specify if total vitamin B12 or active vitamin B12 should be tested. Also, additional testing with MMA and homocysteine is not consistently advised. To date, the most reliable parameter to detect vitamin B12 deficiency is still a matter of debate. According to Herbert's hypothesis, active vitamin B12 is an earlier and more sensitive indicator of vitamin B12 deficiency[36,37], which was supported by later studies[38-41]. Nonetheless, other studies contradict this and state that reliability of active and total vitamin B12 is similar[42,43].

These findings highlight the need for additional research to determine the most optimal parameters and cut-offs to diagnose anaemia caused by nutritional deficiencies.

Laboratory aspects in guidelines

There is limited awareness of laboratory aspects in anaemia guidelines, while all the algorithms use laboratory tests to determine the cause of anaemia. It must be noted that the ferritin, vitamin B12 and folate assays are not harmonised between platforms and there are substantial inter-analyser differences. The lack of harmonisation may result in different values when [illegible] sample is measured in different labs using different analyser platforms[44-47]. Concentrations may also be dependent on the type of material, e.g. serum or plasma that is used for the analysis. In general, serum as well as plasma is acceptable for analysis. Nonetheless, higher or lower values may be found in specific types of material for ferritin, vitamin B12 and folate[40,47,48]. Therefore, if cut-offs are mentioned, the used sample type, analyser platform and method should be specified. Applying inappropriate cut-offs lead to misinterpretation and may cause under- or overdiagnosis of patients. Given the lack of harmonisation of ferritin, vitamin B12 and folate, at this point we recommend referring to local laboratory reference values or cut-offs provided by the local laboratory.

Strengths and limitations

To our knowledge, this is the first study that systematically reviewed anaemia guidelines focusing on the diagnostics of the most common causes of anaemia. In addition to summarising diagnostic strategies, we present recommendations that may be used for developing new guidelines. A limitation of this study is that we only selected guidelines of countries with comparable healthcare systems that were available in Dutch, German or English, potentially missing other relevant guidelines. Nonetheless, our review provides an overview of diagnostic strategies from guidelines from all over the world.

Conclusion

All in all, there was a lot of variation in the included diagnostic criteria for anaemia caused by nutritional deficiencies in guidelines for the adult population. Even when similar diagnostic strategies were used, differences in cut-off values were seen and the supporting evidence was variable. Further research is needed to determine optimal algorithms. Even though laboratory tests are a fundamental aspect of the diagnostic algorithms, description of relevant laboratory aspects was lacking. For future guidelines, it will be important to include relevant laboratory aspects in guidelines, considering appropriate diagnostics and clinical decision limits.

References

1. World Health Organization. Worldwide prevalence of anaemia 1993-2005 - WHO global database on anaemia. Edited by Bruno de Benoist, Erin McLean, Ines Egli and Mary Cogswell (2008).
2. Safiri, S., Kolahi, A.A., Noori, M., Nejadghaderi, S.A., Karamzad, N., Bragazzi, N.L., Sullman, M.J.M., Abdollahi, M., Collins, G.S., Kaufman, J.S., and Grieger, J.A. (2021). Burden of anemia and its underlying causes in 204 countries and territories, 1990-2019: results from the Global Burden of Disease Study 2019. J Hematol Oncol *14*, 185. 10.1186/s13045-021-01202-2.
3. Cascio, M.J., and DeLoughery, T.G. (2017). Anemia: Evaluation and Diagnostic Tests. Med Clin North Am *101*, 263-284. 10.1016/j.mcna.2016.09.003.
4. Kassebaum, N.J., and Collaborators, G.B.D.A. (2016). The Global Burden of Anemia. Hematol Oncol Clin North Am *30*, 247-308. 10.1016/j.hoc.2015.11.002.
5. de Jong, A.M., Bozic, C., Candido, F., van Stralen, K.J., and de Boer, B.A. (2023). [The cause of anemia is underdiagnosed in primary care]. Ned Tijdschr Geneeskd *167*.
6. Zhi, M., Ding, E.L., Theisen-Toupal, J., Whelan, J., and Arnaout, R. (2013). The landscape of inappropriate laboratory testing: a 15-year meta-analysis. PLoS One *8*, e78962. 10.1371/journal.pone.0078962.
7. Kip, M.M.A., Oonk, M.L.J., Levin, M.D., Schop, A., Bindels, P.J.E., Kusters, R., and Koffijberg, H. (2020). Preventing overuse of laboratory diagnostics: a case study into diagnosing anaemia in Dutch general practice. BMC Med Inform Decis Mak *20*, 178. 10.1186/s12911-020-01198-8.
8. Centre for Perioperative Care. Guideline for the Management of Anaemia in the Perioperative Pathway. Available from https://cpoc.org.uk/guidelines-resources-guidelines/anaemia-perioperative-pathway. (2022).
9. Behnisch, W., Muckenthaler, M., and Kulozik, A. (2021). Arbeitsgemeinschaft der Wissenschaftlichen Medizinischen Fachgesellschaften.Eisenmangelanamie. Available from https://register.awmf.org/de/leitlinien/detail/025-021.
10. Snook, J., Bhala, N., Beales, I.L.P., Cannings, D., Kightley, C., Logan, R.P.H., Pritchard, D.M., Sidhu, R., Surgenor, S., Thomas, W., et al. (2021). British Society of Gastroenterology. Guidelines for the Management of Iron Deficiency Anaemia in Adults. Gut *70*, 2030-2051.
11. Bouma, M., Burgers, J.S., Drost, B., Den Elzen, W.P.J., Luchtman, T., Oosterhuis, W.P., Woutersen-Koch, H., and Van Wijk , M. (2021). Nederlands Huisarts Genootschap (NHG)-Standaard Anemie (M76). Available from https://richtlijnen.nhg.org/standaarden/anemie.
12. Ministry of Public Health Qatar. National Clinical Guidelines: The Assessment & Management of Common Anaemias in Adults. Available from https://moph.gov.qa/english/OurServices/eservices/Pages/Clinical-Guidelines.aspx#A. (2020).
13. Ko, C.W., Siddique, S.M., Patel, A., Harris, A., Sultan, S., Altayar, O., and Falck-Ytter, Y. (2020). American Gastroenterological Association. AGA Clinical Practice Guidelines on the Gastrointestinal Evaluation of Iron Deficiency Anemia. Gastroenterology *159*, 1085-1094. doi:10.1053/j.gastro.2020.06.046.
14. Royal College of Nursing. Iron deficiency anaemia in adults. Available from https://www.rcn.org.uk/Professional-Development/publications/pub-007460. (2019).
15. Ministry of Health, British Columbia. Doctors of BC. BCGuidelines. Iron Deficiency - Diagnosis and Management. Available from https://www2.gov.bc.ca/gov/content/health/practitioner-professional-resources/bc-guidelines/iron-deficiency. (2019).
16. Toward Optimized Practice Iron Defiency Anemia Committee. Iron deficiency anemia clinical practice guideline. Edmonton, AB: Toward Optimized Practice. Available from http://www.topalbertadoctors.org. (2018).
17. National Institute for Care and health Excellence (NICE). Iron deficiency anaemia. Available from cks.nice.org.uk. (2021).

18. NHS Greater Glasgow and Clyde. Clinical guideline. Iron deficiency Anaemia in Adults: Oral and Intravenous Iron Therapy Treatment. Available from https://rightdecisions.scot.nhs.uk/m/2295/ida.pdf. (2022).
19. National Institute for Care and health Excellence (NICE). Anaemia - B12 and folate deficiency. Available from cks.nice.org.uk. (2020).
20. Deutsche Gesellschaft fur Anasthesiologie und Intensivmedizin in Kooperation met der Arbeitsgemeinschaft der Wissenschaftlichen Medizinischen Fachgesellschaften. Praoperative Anamie: Diagnostik und Therapie der Praoperative Anamie. (2018).
21. American Medical Directors Association. Anemia in the long-term care setting. Available from https://paltc.org/products/anemia-long-term-care-setting. (2007).
22. World Health Organization. Guideline on haemoglobin cutoffs to define anaemia in individuals and populations. (2024).
23. Fanshawe, T.R., Ordonez-Mena, J.M., Turner, P.J., Bruel, A.V.D., Shine, B., and Hayward, G.N. (2018). Frequencies and patterns of laboratory test requests from general practice: a service evaluation to inform point-of-care testing. J Clin Pathol *71*, 1065-1071. 10.1136/jclinpath-2018-205242.
24. Ursem, S.R., Boelen, A., Bruinstroop, E., Elders, P.J.M., Gussekloo, J., Poortvliet, R.K.E., Heijboer, A.C., and den Elzen, W.P.J. (2024). A systematic review of subclinical hyperthyroidism guidelines: a remarkable range of recommendations. Eur Thyroid J *13*. 10.1530/ETJ-24-0036.
25. Levi, M., Simonetti, M., Marconi, E., Brignoli, O., Cancian, M., Masotti, A., Pegoraro, V., Heiman, F., Cricelli, C., and Lapi, F. (2019). Gender differences in determinants of iron-deficiency anemia: a population-based study conducted in four European countries. Ann Hematol *98*, 1573-1582. 10.1007/s00277-019-03707-w.
26. World Health Organization. WHO guideline on use of ferritin concentrations to assess iron status in individuals and populations. (2020).
27. den Elzen, W.P., Westendorp, R.G., Frolich, M., de Ruijter, W., Assendelft, W.J., and Gussekloo, J. (2008). Vitamin B12 and folate and the risk of anemia in old age: the Leiden 85-Plus Study. Arch Intern Med *168*, 2238-2244. 10.1001/archinte.168.20.2238.
28. den Elzen, W.P., and Gussekloo, J. (2011). Anaemia in older persons. Neth J Med *69*, 260-267.
29. Smelt, A.F., Gussekloo, J., Bermingham, L.W., Allen, E., Dangour, A.D., Eussen, S.J., Favrat, B., De Groot, L.C., Kok, F.J., Kwok, T., et al. (2018). The effect of vitamin B12 and folic acid supplementation on routine haematological parameters in older people: an individual participant data meta-analysis. Eur J Clin Nutr *72*, 785-795. 10.1038/s41430-018-0118-x.
30. Schop, A., Stouten, K., Riedl, J.A., van Houten, R.J., Leening, M.J.G., Bindels, P.J.E., and Levin, M.D. (2021). The accuracy of mean corpuscular volume guided anaemia classification in primary care. Fam Pract *38*, 735-739. 10.1093/fampra/cmab034.
31. Garcia-Casal, M.N., Pasricha, S.R., Martinez, R.X., Lopez-Perez, L., and Pena-Rosas, J.P. (2018). Are Current Serum and Plasma Ferritin Cut-offs for Iron Deficiency and Overload Accurate and Reflecting Iron Status? A Systematic Review. Arch Med Res *49*, 405-417. 10.1016/j.arcmed.2018.12.005.
32. Garcia-Casal, M.N., Pasricha, S.R., Martinez, R.X., Lopez-Perez, L., and Pena-Rosas, J.P. (2021). Serum or plasma ferritin concentration as an index of iron deficiency and overload. Cochrane Database Syst Rev *5*, CD011817. 10.1002/14651858.CD011817.pub2.
33. Peyrin-Biroulet, L., Williet, N., and Cacoub, P. (2015). Guidelines on the diagnosis and treatment of iron deficiency across indications: a systematic review. Am J Clin Nutr *102*, 1585-1594. 10.3945/ajcn.114.103366.
34. Raghavan, R., Ashour, F.S., and Bailey, R. (2016). A Review of Cutoffs for Nutritional Biomarkers. Adv Nutr *7*, 112-120. 10.3945/an.115.009951.

35. Aparicio-Ugarriza, R., Palacios, G., Alder, M., and Gonzalez-Gross, M. (2015). A review of the cut-off points for the diagnosis of vitamin B12 deficiency in the general population. Clin Chem Lab Med *53*, 1149-1159. 10.1515/cclm-2014-0784.
36. Herbert, V. (1987). The 1986 Herman award lecture. Nutrition science as a continually unfolding story: the folate and vitamin B-12 paradigm. Am J Clin Nutr *46*, 387-402. 10.1093/ajcn/46.3.387.
37. Herbert, V. (1994). Staging vitamin B-12 (cobalamin) status in vegetarians. Am J Clin Nutr *59*, 1213S-1222S. 10.1093/ajcn/59.5.1213S.
38. Heil, S.G., de Jonge, R., de Rotte, M.C., van Wijnen, M., Heiner-Fokkema, R.M., Kobold, A.C., Pekelharing, J.M., Adriaansen, H.J., Sanders, E., Trienekens, P.H., et al. (2012). Screening for metabolic vitamin B12 deficiency by holotranscobalamin in patients suspected of vitamin B12 deficiency: a multicentre study. Ann Clin Biochem *49*, 184-189. 10.1258/acb.2011.011039.
39. Jarquin Campos, A., Risch, L., Nydegger, U., Wiesner, J., Vazquez Van Dyck, M., Renz, H., Stanga, Z., and Risch, M. (2020). Diagnostic Accuracy of Holotranscobalamin, Vitamin B12, Methylmalonic Acid, and Homocysteine in Detecting B12 Deficiency in a Large, Mixed Patient Population. Dis Markers *2020*, 7468506. 10.1155/2020/7468506.
40. Nexo, E., and Hoffmann-Lucke, E. (2011). Holotranscobalamin, a marker of vitamin B-12 status: analytical aspects and clinical utility. Am J Clin Nutr *94*, 359S-365S. 10.3945/ajcn.111.013458.
41. Valente, E., Scott, J.M., Ueland, P.M., Cunningham, C., Casey, M., and Molloy, A.M. (2011). Diagnostic accuracy of holotranscobalamin, methylmalonic acid, serum cobalamin, and other indicators of tissue vitamin B(1)(2) status in the elderly. Clin Chem *57*, 856-863. 10.1373/clinchem.2010.158154.
42. Golding, P.H. (2016). Holotranscobalamin (HoloTC, Active-B12) and Herbert's model for the development of vitamin B12 deficiency: a review and alternative hypothesis. Springerplus *5*, 668. 10.1186/s40064-016-2252-z.
43. Remacha, A.F., Sarda, M.P., Canals, C., Queralto, J.M., Zapico, E., Remacha, J., and Carrascosa, C. (2014). Role of serum holotranscobalamin (holoTC) in the diagnosis of patients with low serum cobalamin. Comparison with methylmalonic acid and homocysteine. Ann Hematol *93*, 565-569. 10.1007/s00277-013-1905-z.
44. Hoofnagle, A.N. (2017). Harmonization of blood-based indicators of iron status: making the hard work matter. Am J Clin Nutr *106*, 1615S-1619S. 10.3945/ajcn.117.155895.
45. Devalia, V., Hamilton, M.S., Molloy, A.M., and British Committee for Standards in, H. (2014). Guidelines for the diagnosis and treatment of cobalamin and folate disorders. Br J Haematol *166*, 496-513. 10.1111/bjh.12959.
46. Choy, K.W., Sezgin, G., Wijeratne, N., Calleja, J., Liwayan, R., Rathnayake, G., McFarlane, R., McNeil, A., Doery, J.C.G., Lu, Z., et al. (2022). Assessment of analytical bias in ferritin assays and impact on functional reference limits. Pathology *54*, 302-307. 10.1016/j.pathol.2021.06.123.
47. Owen, W.E., and Roberts, W.L. (2003). Comparison of five automated serum and whole blood folate assays. Am J Clin Pathol *120*, 121-126. 10.1309/L2U6-HH5K-AYG4-8L40.
48. Ji, Y., Faddy, H., Hyland, C., and Flower, R. (2015). A plasma ferritin is not always a serum ferritin. Pathology *47* (*1*), S89-S90.
49. Munoz, M., Acheson, A.G., Auerbach M., Besser, M., Habler, O., Kehlet, H., Liumbruno, G.M., et al. (2017). International consensus statement on the peri-operative management of anaemia and iron deficiency. Anaesthesia. *72(2)*, 233-247. 10.1111/anae.13773.

Table 1. Summarized haemoglobin cut-offs in the included guidelines to define anaemia

Guideline	Year	Country	Haemoglobin level	Evidence	Reference
NHS Greater Glasgow and Clyde	2022	UK	M <13 g/dL, F <11.5 g/dL	1	18
Centre for Perioperative Care	2022	UK	M <13 g/dL, F <12 g/dL *	4	8
Association of Scientific Medical Societies Germany	2021	Germany	M <13 g/dL, F <12 g/dL	4	9
British Society of Gastroenterology	2021	UK	< N	NA	10
National Institute for Health and Care Excellence	2021	UK	M <13 g/dL, F <12 g/dL	4	17
Dutch College of General Practitioners	2021	th[illegible] Netherlands	M < 13 g/dL, F <12 g/dL #	4	11
Ministry of Public Health Qatar	2020	Qatar	M <13 g/dL, F <12 g/dL	4	12
American Gastroenterological Association	2020	USA	M <13 g/dL, F <12 g/dL	4	13
National Institute for Health and Care Excellence	2020	UK	M <13 g/dL, F <12 g/dL #	4	19
Royal College of Nursing	2019	UK	M <13 g/dL, F <12 g/dL	4	14
BC guidelines	2019	Canada	< N	NA	15
Association of Scientific Medical Societies Germany	2018	Germany	M <13 g/dL, F <12 g/dL, Drop > 2 g/dL	4	20
Toward Optimized Practice	2018	Canada	M <13.5 g/dL, F <12 g/dL #	1	16
American Medical Directors Association	2007	USA	< N	NA	21

* international consensus state[illegible]t: Hb <13 g/dL for both sexes (ref. 49)
# Suggested cut-offs: use local laboratory cut-off values for assessment

Table 2. Summarized diagnostic algorithms of the included iron deficiency anaemia guidelines.

Guideline	Year	Country	MCV (fl)	Ferritin (µg/L)	Iron	Transferrin	Transferrin sat (%)	TIBC	CRP (mg/L) or ESR	Ret-Hb / Reticulocytes	Evidence	Reference
NHS Greater Glasgow and Clyde	2022	UK		< 15 or 15 - 50 †							1	18
Centre for Perioperative Care	2022	UK		< 30 * or 30 - 100 †			<20% *		> 5 †	< 30 pg *	3	8
Association of Scientific Medical Societies Germany	2021	Germany	< 78	M < 35, F <23 or ≥ N †						< 28 pg / ≤ N	2	9
British Society of Gastroenterology	2021	UK	< N*	< N or N *			Low *		> N *	< N *	4	10
National Institute for Health and Care Excellence	2021	UK		< 30 ‡							3	17
Dutch College of General Practitioners	2021	the Netherlands	≤ N	< N or < 100 † *	< N *	> N *					2	11
Ministry of Public Health Qatar	2020	Qatar	< N	≤ N ‡	< N			≥ N			3	12
American Gastroenterological Association	2020	USA		< 45 ‡							4	13
Royal College of Nursing	2019	UK		< 30 or 30 - 100 ^			< 20% ^		> 5 ^		1	14
BC guidelines	2019	Canada		< 15 or 15 - 30 $ or < 100 †							2	15
Association of Scientific Medical Societies Germany	2018	Germany	< N	< 30 * or 30 - 100 * or > 300 †#			< 20% * or †#				3	20
Toward Optimized Practice	2018	Canada	< 75 *	M < 30 *, F < 13 or < 100 †							3	16
American Medical Directors Association	2007	USA	< N	< N or < N # or N †	< N # †	> N #	< N # †	> N #			2	21

* and/or
# and
^ or
† Patients with chronic (inflammatory) disease, active inflammation or malignancy
‡ Ferritin is an acute phase protein and increases in patients with infection or inflammation
$ Probably iron deficiency

Table 3. Summarized diagnostic algorithms of the included vitamin B12 / folate deficiency anaemia guidelines.

				Vitamin B12				Folate				Folate and vit. B12			
**Guideline**	**Year**	**Country**	**MCV (fl)**	**Vit. B12 (pmol/L)**	**Active vit. B12**	**MMA**	**Homocysteine**	**Folate (nmol/L)**	**RBC folate (nmol/L)**	**MMA**	**Homocysteine**	**LDH**	**Reticulocytes**	**Evidence**	**Reference**
Centre for Perioperative Care	2022	UK	≥ N	< N				< N						3	8
Dutch College of General Practitioners	2021	the Netherlands	≥ N	< N or low N *		Optional (1st) *	Optional (2nd) *	< N				> N	< N	3	11
National Institute for Health and Care Excellence	2020	UK	≥ N	< 148				< 7 or N #	< 340 #					3	19
Ministry of Public Health Qatar	2020	Qatar	> N	< N		> N		< N		N				3	12
Association of Scientific Medical Societies Germany	2018	Germany	> N	< N or low N *	Optional *	Optional *	Optional *	< N ^	Confirmation		Optional			3	18
American Medical Directors Association	2007	USA	≥ N	< N		> N	> N	< N						2	21

* and / or
# with normal vit. B12
^ related to food intake, a second fasting blood test is required

Figure 1. Flowchart of selected guidelines

Figure 2. Observations in current guidelines and recommendations for future guideline revisions with respect to anaemia

Figure 1

Click here to access/download;Figure;Roemer_denElzen_Figure 1_Flowchart of selected guidelines.pdf

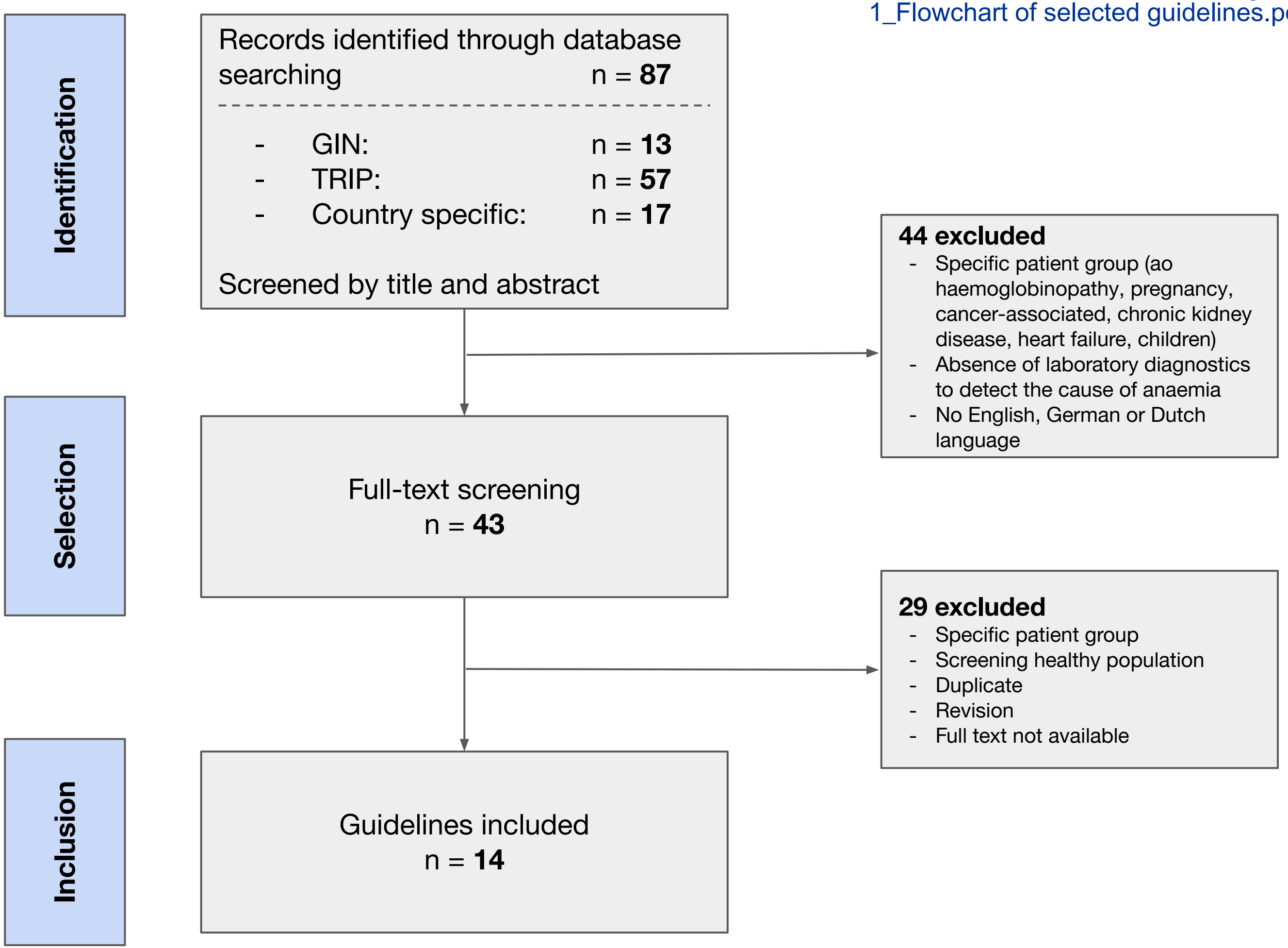

Figure 2

Click here to access/download;Figure;Roemer_denElzen_Figure 2_Recommendations.pdf

Observation in current guidelines	Recommendation for future guidelines
1. Plethora of different anaemia guidelines per organ, patient group and underlying disease for primary and secondary care	1. Develop guidelines specifically for the primary and secondary care settings and align these
2. No specific attention is paid to individual subgroups, such as age and sex	2. Include aspects of diversity (at least age and sex) in diagnostic algorithms
3. Variation in algorithms in diagnostic criteria and cut-off values	3. Further research is needed to determine optimal algorithm
4. Most guidelines focus on inclusion of MCV	4. MCV is not the most important parameter, use as supporting parameter
5. Variation in used cut-off values for ferritin	5. The test for ferritin is not harmonized: refer to local laboratory reference ranges instead of mentioning actual cut-off values
6. Variable views on how to deal with inflammation in patients with iron deficiency	6. Mention the importance of acknowledging the presence of inflammation, especially for ferritin
7. Not clear if total or active vitamin B12 should be used	7. Align the recommended test for vitamin B12 deficiency
8. Lack of and/or variable quality of evidence	8. Critically evaluate quality of evidence to include in guideline: rigorous methodology and well-defined approach
9. Little to no awareness of laboratory aspects in guidelines<br>a. Different platforms produce different results<br>b. No harmonization / standardization of tests	9. Specifically mention laboratory aspects in guidelines<br>a. Specify the platform that is used to determine cut-offs<br>b. When there is no harmonization / standardization of the test: refer to local laboratory reference ranges

Click here to access/download
Supporting Information
2025 PRISMA_2020_abstract_checklist.docx

PRISMA checklist

Click here to access/download
Supporting Information
2025 PRISMA_2020_checklist.docx
