# Supplementary material for: Comparison of diagnostic algorithms used in guidelines on nutritional anaemias in adults
Source: PLoS One. 2025 Oct 3;20(10):e0333688. doi: 10.1371/journal.pone.0333688 (PMC12494270; doi:10.1371/journal.pone.0333688)
Supplement: Attachment — Submitted filename: Response to Reviewers_guidelines on anaemia.docx [file pone.0333688.s007.docx]

Dear dr. Mayne,

Thank you for the evaluation of our manuscript: **Diagnostic algorithms in guidelines on anaemia: a systematic review**. We appreciate your and the reviewers’ careful assessment and the thoughtful comments regarding the work. We carefully evaluated each point and believe that addressing these comments helped us to improve the quality of the manuscript.

All modifications have been highlighted in the marked-up copy of our manuscript. Below please find a point-by-point response to your and the reviewers’ comments and journal requirements.

Sincerely,

Anne Margreet de Jong

Laboratory specialist

**Comments of academic editor**

The reviewers suggested some minor amendments including some grammatical changes and in addition a few references to support the ferritin analysis. I also feel that the proposed title change  (review of nutritional anaemias) is a good suggestion.

Thank you for the evaluation of our manuscript. We agree with the minor amendments and proposed title change and have adjusted the manuscript accordingly.

**Review comments to the Author**

Reviewer #1: Thank you for the opportunity to review your manuscript that investigates similarities and differences in diagnostic algorithms of clinical treatment guidelines of nutritional anaemia in adult patients.

Major observations:

1. While many of the PRISMA 2020 checklist items were followed, I consider this manuscript to represent a narrative and thematic synthesis describing and comparing the guidelines and their recommendations. The quality of evidence and strength of recommendations contained in each clinical practice guideline were not evaluated using a recognised approach e.g., a rapid (iCAHE) or a complex instrument (the GRADE, AGREE II and AGREE-REX). In this respect I consider the methodology to fall short of a complete systematic review. I question the application of the scoring system used in the manuscript to rate the quality of the guidelines (p.5 3rd paragraph, 2^nd^ sentence) as a suitable alternative. Table 1 shows three CPGs (8[2022], 14[2019], and 20[2018]) with similar diagnostic algorithms for iron deficiency yet variable evidence.

We acknowledge the lack of a recognized grading approach for the different guidelines. However, it is important to note that we did not evaluate guidelines as a whole, but specifically focused on the laboratory parameters within the guidelines and the evidence that was used to support the different diagnostic algorithms. Consequently, the same guideline may have a different quality for the different nutritional anaemias, e.g. iron deficiency anaemia (evidence = 2) and vitamin B12/folate deficiency anaemia (evidence = 3) in the Dutch anaemia guideline. Similarly, even though a similar diagnostic algorithm is used in different guidelines, the evidence supporting this algorithm may differ. To clarify this, we changed the following sentence in the discussion on page 10, line 10: “Furthermore, a lot of variation was seen in the recommended diagnostic criteria, cut-off values and evidence underpinning the algorithms, even in the presence of similar diagnostic strategies.”

We do agree that the wording in our title: “systematic review” may be misleading. Therefore, we removed this from the title and changed it to: “Comparison of diagnostic algorithms in guidelines on nutritional anaemias in adults”.

Minor observations:

1. I believe the title of the manuscript should be amended to reflect the focus of the work: e.g.,

Diagnostic algorithms in guidelines on nutritional anaemias in adults.

Thank you, we agree with this suggestion and have adjusted the title to: “Comparison of diagnostic algorithms in guidelines on nutritional anaemias in adults”.

2. While Qatar’s health care system is a leader in the Gulf Cooperation Council, is it really similar to the healthcare systems of e.g., Western European countries in this study? Developing vs. developed health care system? As an example, in 2020 Qatar’s health care spending has exceeded its European counterparts but it was predominantly toward secondary care (70-75%), with ~20-25% for primary care and <5% for self-care. The comparative breakdown for the Netherlands was ~45%, ~45%, and ~10% for secondary, primary, and self-care, respectively.

There is certainly a difference between health care systems in different countries, but this is true even within Western European countries. For instance: in the Dutch health care system, the general practitioner acts as a gatekeeper for referral to medical specialists, while in the German health care system, patients have direct access to medical specialists. Qatar has consistently ranked among the top healthcare systems globally and we therefore believe that it is justified to include the Qatar anaemia guideline in this manuscript.

3. p.4. 2nd paragraph 2nd last sentence: amend under- of overtreatment to under- or overtreatment

Done.

4. p.5 3rd paragraph 1st and 3rd sentence: repetitive: To ensure effective and standardised care, anaemia guidelines have been developed. Adhering to established clinical guidelines is advised to ensure effective and standardised care. Suggest consolidation.

Thank you for pointing this out. We removed the second sentence.

5. p.5 3rd paragraph 4th sentence: I am unsure how physiological needs vary by ethnicity. Reconsider or substantiate.

Agree. We reworded the sentence to: “For different physiological settings, including age, sex and pregnancy status or for specific patient groups, different guidelines for diagnosing anaemia have been developed.”

6. References should be extensively reviewed to comply with the journal’s author guideline. In particular; 1. Use the Vancouver referencing style and, for consistency, restrict authors to the first six followed by et al. and 2. Review referencing style of internet resources. Some of the listed URLs are stale.

Done, see also point 6 of journal requirements.

7. Abbreviations used in the tables should be explained in the caption e.g., 8. The ferritin cut-off of reference 9 in Table 2 appears erroneous.

We added a caption explaining the abbreviations to the tables.

Reviewer #2: This is a highly relevant and useful article which is well-written.

Some minor grammatical edits are required, and additional suggested references are provided to include important pre-analytical factors affecting serum ferritin levels which are not widely recognized and can lead to diagnostic confusion.

We would like to thank the reviewer for her/his appreciative comments and for the effort invested in evaluating our work. We incorporated the suggested edits in the attached document. See specifics below:

Edits in introduction:

- grammar: to find the underlying cause
- grammar: from ferritin only, to ferritin in various combinations...
- suggest reword: affecting up to 25%...
- add: red blood cells for the age and sex of the individual.
- reword: The diagnosis of anaemia typically includes ...
- suggest reword: physiological settings including age, sex, ... different guidelines for the diagnosis of anaemia have been developed.

Edits in discussion:

- the CBC
- suggest delete.

reword: we recommend including at least age and sex in the diagnostic algorithms

- when the same sample is measured

Edits in Table 1:

- The (capitalise)
- This international statement Hb specifically refers to the pre-operative management of anaemia. Please add this into the legend:

* International consensus statement for pre-operative anaemia: Hb< 13 g/dl for both sexes.

Edits in Figure 2:

- use MCV as a supportive parameter only

Furthermore, we incorporated the suggested references in the text.

Specifically, on page 10, we added the reference to the ferritin cut-off study to the following sentence on line 30: “For iron deficiency anaemia, ferritin was included in all algorithms. However, the used cut-offs were variable, as has been previously described”

In addition, we added the following sentence to the discussion, page 10, lines 35-38:

“Furthermore, ferritin levels are affected by recent oral iron supplementation intake, coffee and other dietary factors. Therefore, it is recommended to fast and withhold supplements before measuring ferritin. Nonetheless, these factors are not emphasized in guidelines, and therefore this can lead to diagnostic confusion.”

**Journal requirements**

When submitting your revision, we need you to address these additional requirements.

1. Please ensure that your manuscript meets PLOS ONE's style requirements, including those for file naming.

Done.

2. We note that your Data Availability Statement is currently as follows: [All relevant data are within the manuscript and its Supporting Information files.]

Please confirm at this time whether or not your submission contains all raw data required to

replicate the results of your study.

Submission contains all data required.

If there are ethical or legal restrictions on sharing a de-identified data set, please explain them in detail (e.g., data contain potentially sensitive information, data are owned by a third-party organization, etc.) and who has imposed them (e.g., an ethics committee). Please also provide contact information for a data access committee, ethics committee, or other institutional body to which data requests may be sent. If data are owned by a third party, please indicate how others may request data access.

There are no ethical or legal restrictions.

3. Please include a copy of Table 1, 2 & 3 which you refer to in your text on page 7.

Tables are placed in the manuscript file directly after the paragraph in which it is first cited, as described in the journal requirements.

4. Please include captions for your Supporting Information files at the end of your manuscript, and update any in-text citations to match accordingly. Please see our Supporting Information guidelines for more information: http://journals.plos.org/plosone/s/supporting-information.

Supporting Information files are updated.

**Supporting information**

Supplementary Table 1. Exclusion table

Supplementary Table 2. PRISMA abstract checklist

Supplementary Table 3. PRISMA checklist

5. As required by our policy on Data Availability, please ensure your manuscript or supplementary information includes the following:

A numbered table of all studies identified in the literature search, including those that were excluded from the analyses. For every excluded study, the table should list the reason(s) for exclusion.

If any of the included studies are unpublished, include a link (URL) to the primary source or detailed information about how the content can be accessed.

A table of all data extracted from the primary research sources for the systematic review and/or meta-analysis. The table must include the following information for each study:

Name of data extractors and date of data extraction

Confirmation that the study was eligible to be included in the review.

All data extracted from each study for the reported systematic review and/or meta-analysis that would be needed to replicate your analyses.

If data or supporting information were obtained from another source (e.g. correspondence with the author of the original research article), please provide the source of data and dates on which the data/information were obtained by your research group.

We generated a table containing all identified guidelines in our database search. The dates of the search and date of assessment and consensus was added. Furthermore, we added the reason for exclusion. See Supplementary Table 1.

If applicable for your analysis, a table showing the completed risk of bias and quality/certainty assessments for each study or outcome. Please ensure this is provided for each domain or parameter assessed. For example, if you used the Cochrane risk-of-bias tool for randomized trials, provide answers to each of the signalling questions for each study. If you used GRADE to assess certainty of evidence, provide judgements about each of the quality of evidence factor. This should be provided for each outcome.

Not applicable

An explanation of how missing data were handled.

Not applicable

This information can be included in the main text, supplementary information, or relevant data repository. Please note that providing these underlying data is a requirement for publication in this journal, and if these data are not provided your manuscript might be rejected.

6. Please review your reference list to ensure that it is complete and correct. If you have cited papers that have been retracted, please include the rationale for doing so in the manuscript text, or remove these references and replace them with relevant current references. Any changes to the reference list should be mentioned in the rebuttal letter that accompanies your revised manuscript. If you need to cite a retracted article, indicate the article’s retracted status in the References list and also include a citation and full reference for the retraction notice.

Done, reference list was updated.
