# Supplementary material for: Comparison of diagnostic algorithms used in guidelines on nutritional anaemias in adults
Source: PLoS One. 2025 Oct 3;20(10):e0333688. doi: 10.1371/journal.pone.0333688 (PMC12494270; doi:10.1371/journal.pone.0333688)
Supplement: Attachment — Submitted filename: PONE-D-25-00163_guidelines_anaemias_R2JJO.docx [file pone.0333688.s006.docx]

**PLOS ONE**

### Comparison of diagnostic algorithms used in guidelines on nutritional anaemias in adults

--Manuscript Draft--

| **Manuscript Number:** | PONE-D-25-00163R1 |
| --- | --- |
| **Article Type:** | Research Article |
| **Full Title:** | Comparison of diagnostic algorithms used in guidelines on nutritional anaemias in adults |
| **Short Title:** | Diagnostic algorithms in anaemias |
| **Corresponding Author:** | Anne Margreet De Jong, PhD Atalmedial diagnostic centre  Amsterdam, NETHERLANDS, KINGDOM OF THE |
| **Keywords:** | general practice, anaemia, iron deficiency, ferritin, vitamin B12 deficiency, folate deficiency, guidelines, laboratory aspects |
| **Abstract:** | Background  Anaemia is highly prevalent and commonly caused by nutritional deficiencies. Guidelines often include an algorithm to find the underlying cause. Here, we compared diagnostic algorithms and suggested laboratory tests for anaemia in clinical practice in countries with similar healthcare systems, focusing on iron, vitamin B12 and folate deficiency in the general adult population.  Methods  We searched for guidelines on anaemia in the Trip, Guidelines International Network and country specific databases. To be selected for this systematic review, the guidelines had to include diagnostic criteria or algorithms to determine the cause of anaemia in the general adult population.  Results  In total, 14 records were included. For iron deficiency anaemia, guidelines varied in diagnostic criteria ranging from use of ferritin only, to ferritin in various combinations with a variety of other parameters, with different cut-off values. For vitamin B12 or folate deficiency, besides measurement of vitamin B12 or folate, some guidelines mentioned methylmalonic acid or homocysteine. Quality of evidence underlying cut-offs and parameters was variable, and laboratory aspects were underrepresented.  Conclusions  There was greatvariation in the included diagnostic algorithms, especially for iron deficiency anaemia. Differences in cut-off values were seen, even when using similar diagnostic strategies. Furthermore, supporting evidence was variable. Further research is needed to determine optimal algorithms. Our findings highlight the need for inclusion of relevant laboratory aspects in guidelines, appropriate diagnostics and clinical decision limits. |
| **Order of Authors:** | Margaretha G.M. Roemer, PhD |
|  | Wendy P.J. den Elzen, PhD |
|  | Rosalinde K.E. Poortvliet, PhD |
|  | Jacobijn Gussekloo, Prof. Dr. |
|  | Steef Kurstjens, PhD |
|  | Bauke A. de Boer, PhD |
|  | Anne Margreet De Jong, PhD |
| **Response to Reviewers:** | Comments of academic editor  The reviewers suggested some minor amendments including some grammatical changes and in addition a few references to support the ferritin analysis. I also feel that the proposed title change (review of nutritional anaemias) is a good suggestion.  Thank you for the evaluation of our manuscript. We agree with the minor amendments |

|  | and proposed title change and have adjusted the manuscript accordingly. Review comments to the Author  Reviewer #1: Thank you for the opportunity to review your manuscript that investigates similarities and differences in diagnostic algorithms of clinical treatment guidelines of nutritional anaemia in adult patients.  Major observations:   1. While many of the PRISMA 2020 checklist items were followed, I consider this manuscript to represent a narrative and thematic synthesis describing and comparing the guidelines and their recommendations. The quality of evidence and strength of recommendations contained in each clinical practice guideline were not evaluated using a recognised approach e.g., a rapid (iCAHE) or a complex instrument (the GRADE, AGREE II and AGREE-REX). In this respect I consider the methodology to fall short of a complete systematic review. I question the application of the scoring system used in the manuscript to rate the quality of the guidelines (p.5 3rd paragraph, 2nd sentence) as a suitable alternative. Table 1 shows three CPGs (8[2022], 14[2019], and 20[2018]) with similar diagnostic algorithms for iron deficiency yet variable evidence.   We acknowledge the lack of a recognized grading approach for the different guidelines. However, it is important to note that we did not evaluate guidelines as a whole, but specifically focused on the laboratory parameters within the guidelines and the evidence that was used to support the different diagnostic algorithms.  Consequently, the same guideline may have a different quality for the different nutritional anaemias, e.g. iron deficiency anaemia (evidence = 2) and vitamin B12/folate deficiency anaemia (evidence = 3) in the Dutch anaemia guideline. Similarly, even though a similar diagnostic algorithm is used in different guidelines, the evidence supporting this algorithm may differ. To clarify this, we changed the following sentence in the discussion on page 10, line 10: “Furthermore, a lot of variation was seen in the recommended diagnostic criteria, cut-off values and evidence underpinning the algorithms, even in the presence of similar diagnostic strategies.”  We do agree that the wording in our title: “systematic review” may be misleading. Therefore, we removed this from the title and changed it to: “Comparison of diagnostic algorithms in guidelines on nutritional anaemias in adults”.  Minor observations:   1. I believe the title of the manuscript should be amended to reflect the focus of the work: e.g.,   Diagnostic algorithms in guidelines on nutritional anaemias in adults.  Thank you, we agree with this suggestion and have adjusted the title to: “Comparison of diagnostic algorithms in guidelines on nutritional anaemias in adults”.   2. While Qatar’s health care system is a leader in the Gulf Cooperation Council, is it really similar to the healthcare systems of e.g., Western European countries in this study? Developing vs. developed health care system? As an example, in 2020 Qatar’s health care spending has exceeded its European counterparts but it was predominantly toward secondary care (70-75%), with ~20-25% for primary care and   <5% for self-care. The comparative breakdown for the Netherlands was ~45%, ~45%, and ~10% for secondary, primary, and self-care, respectively.  There is certainly a difference between health care systems in different countries, but this is true even within Western European countries. For instance: in the Dutch health care system, the general practitioner acts as a gatekeeper for referral to medical specialists, while in the German health care system, patients have direct access to medical specialists. Qatar has consistently ranked among the top healthcare systems globally and we therefore believe that it is justified to include the Qatar anaemia guideline in this manuscript.   3. p.4. 2nd paragraph 2nd last sentence: amend under- of overtreatment to under- or overtreatment |
| --- | --- |

|  | Done.   4. p.5 3rd paragraph 1st and 3rd sentence: repetitive: To ensure effective and standardised care, anaemia guidelines have been developed. Adhering to established clinical guidelines is advised to ensure effective and standardised care. Suggest consolidation.   Thank you for pointing this out. We removed the second sentence.   5. p.5 3rd paragraph 4th sentence: I am unsure how physiological needs vary by ethnicity. Reconsider or substantiate.   Agree. We reworded the sentence to: “For different physiological settings, including age, sex and pregnancy status or for specific patient groups, different guidelines for diagnosing anaemia have been developed.”   6. References should be extensively reviewed to comply with the journal’s author guideline. In particular; 1. Use the Vancouver referencing style and, for consistency, restrict authors to the first six followed by et al. and 2. Review referencing style of internet resources. Some of the listed URLs are stale.   Done, see also point 6 of journal requirements.   7. Abbreviations used in the tables should be explained in the caption e.g., 8. The ferritin cut-off of reference 9 in Table 2 appears erroneous.   We added a caption explaining the abbreviations to the tables.  Reviewer #2: This is a highly relevant and useful article which is well-written.  Some minor grammatical edits are required, and additional suggested references are provided to include important pre-analytical factors affecting serum ferritin levels which are not widely recognized and can lead to diagnostic confusion.  We would like to thank the reviewer for her/his appreciative comments and for the effort invested in evaluating our work. We incorporated the suggested edits in the attached document. See specifics below:  Edits in introduction:  •grammar: to find the underlying cause  •grammar: from ferritin only, to ferritin in various combinations...  •suggest reword: affecting up to 25%...  •add: red blood cells for the age and sex of the individual.  •reword: The diagnosis of anaemia typically includes ...  •suggest reword: physiological settings including age, sex, ... different guidelines for the diagnosis of anaemia have been developed.  Edits in discussion:  •the CBC  •suggest delete.  reword: we recommend including at least age and sex in the diagnostic algorithms  •when the same sample is measured  Edits in Table 1:  •The (capitalise)  •This international statement Hb specifically refers to the pre-operative management of anaemia. Please add this into the legend:  * International consensus statement for pre-operative anaemia: Hb< 13 g/dl for both sexes.  Edits in Figure 2:  •use MCV as a supportive parameter only  Furthermore, we incorporated the suggested references in the text.  Specifically, on page 10, we added the reference to the ferritin cut-off study to the |
| --- | --- |

|  | following sentence on line 30: “For iron deficiency anaemia, ferritin was included in all algorithms. However, the used cut-offs were variable, as has been previously described”  In addition, we added the following sentence to the discussion, page 10, lines 35-38: “Furthermore, ferritin levels are affected by recent oral iron supplementation intake, coffee and other dietary factors. Therefore, it is recommended to fast and withhold supplements before measuring ferritin. Nonetheless, these factors are not emphasized in guidelines, and therefore this can lead to diagnostic confusion.”  Journal requirements  When submitting your revision, we need you to address these additional requirements.   1. Please ensure that your manuscript meets PLOS ONE's style requirements, including those for file naming.   Done.   2. We note that your Data Availability Statement is currently as follows: [All relevant data are within the manuscript and its Supporting Information files.]   Please confirm at this time whether or not your submission contains all raw data required to  replicate the results of your study. Submission contains all data required.  If there are ethical or legal restrictions on sharing a de-identified data set, please explain them in detail (e.g., data contain potentially sensitive information, data are owned by a third-party organization, etc.) and who has imposed them (e.g., an ethics committee). Please also provide contact information for a data access committee, ethics committee, or other institutional body to which data requests may be sent. If data are owned by a third party, please indicate how others may request data access.  There are no ethical or legal restrictions.   3. Please include a copy of Table 1, 2 & 3 which you refer to in your text on page 7.   Tables are placed in the manuscript file directly after the paragraph in which it is first cited, as described in the journal requirements.   4. Please include captions for your Supporting Information files at the end of your manuscript, and update any in-text citations to match accordingly. Please see our Supporting Information guidelines for more information: [http://journals.plos.org/plosone/s/supporting-information.](http://journals.plos.org/plosone/s/supporting-information)   Supporting Information files are updated.  Supporting information  Supplementary Table 1. Exclusion table Supplementary Table 2. PRISMA abstract checklist Supplementary Table 3. PRISMA checklist   5. As required by our policy on Data Availability, please ensure your manuscript or supplementary information includes the following:   A numbered table of all studies identified in the literature search, including those that were excluded from the analyses. For every excluded study, the table should list the reason(s) for exclusion.  If any of the included studies are unpublished, include a link (URL) to the primary source or detailed information about how the content can be accessed.  A table of all data extracted from the primary research sources for the systematic review and/or meta-analysis. The table must include the following information for each study: |
| --- | --- |

|  | Name of data extractors and date of data extraction  Confirmation that the study was eligible to be included in the review.  All data extracted from each study for the reported systematic review and/or meta- analysis that would be needed to replicate your analyses.  If data or supporting information were obtained from another source (e.g. correspondence with the author of the original research article), please provide the source of data and dates on which the data/information were obtained by your research group.  We generated a table containing all identified guidelines in our database search. The dates of the search and date of assessment and consensus was added. Furthermore, we added the reason for exclusion. See Supplementary Table 1.  If applicable for your analysis, a table showing the completed risk of bias and quality/certainty assessments for each study or outcome. Please ensure this is provided for each domain or parameter assessed. For example, if you used the Cochrane risk-of-bias tool for randomized trials, provide answers to each of the signalling questions for each study. If you used GRADE to assess certainty of evidence, provide judgements about each of the quality of evidence factor. This should be provided for each outcome.  Not applicable  An explanation of how missing data were handled. Not applicable  This information can be included in the main text, supplementary information, or relevant data repository. Please note that providing these underlying data is a requirement for publication in this journal, and if these data are not provided your manuscript might be rejected.  6. Please review your reference list to ensure that it is complete and correct. If you have cited papers that have been retracted, please include the rationale for doing so in the manuscript text, or remove these references and replace them with relevant current references. Any changes to the reference list should be mentioned in the rebuttal letter that accompanies your revised manuscript. If you need to cite a retracted article, indicate the article’s retracted status in the References list and also include a citation and full reference for the retraction notice.  Done, reference list was updated. |
| --- | --- |
| **Additional Information:** |  |
| **Question** | **Response** |
| **Financial Disclosure**  Enter a financial disclosure statement that describes the sources of funding for the work included in this submission. Review the [submission guidelines](http://journals.plos.org/plosone/s/submission-guidelines#loc-financial-disclosure-statement) for detailed requirements. View published research articles from [*PLOS ONE*](https://journals.plos.org/plosone/) for specific examples.  This statement is required for submission and **will appear in the published article** if the submission is accepted. Please make sure it is accurate. | The author(s) received no specific funding for this work. |

| **Funded studies**  Enter a statement with the following details:   - Initials of the authors who received each award - Grant numbers awarded to each author - The full name of each funder - URL of each funder website - Did the sponsors or funders play any role in the study design, data collection and analysis, decision to publish, or preparation of the manuscript? |  |
| --- | --- |
| Did you receive funding for this work? |  |
| **Competing Interests**  Use the instructions below to enter a competing interest statement for this submission. On behalf of all authors, disclose any [competing interests](http://journals.plos.org/plosone/s/competing-interests) that could be perceived to bias this work—acknowledging all financial support and any other relevant financial or non- financial competing interests.  This statement is required for submission and **will appear in the published article** if the submission is accepted. Please make sure it is accurate and that any funding sources listed in your Funding Information later in the submission form are also declared in your Financial Disclosure statement.  View published research articles from  [*PLOS ONE*](http://journals.plos.org/plosone/) for specific examples. | The authors have declared that no competing interests exist. |

| **NO authors have competing interests**  Enter: *The authors have declared that no competing interests exist.*  **Authors with competing interests**  Enter competing interest details beginning with this statement:  *I have read the journal's policy and the authors of this manuscript have the following competing interests: [insert competing interests here]* |  |
| --- | --- |
| * typeset |  |
| **Ethics Statement**  Enter an ethics statement for this submission. This statement is required if the study involved:   - Human participants - Human specimens or tissue - Vertebrate animals or cephalopods - Vertebrate embryos or tissues - Field research   Write "N/A" if the submission does not require an ethics statement.  General guidance is provided below. Consult the [submission guidelines](http://journals.plos.org/plosone/s/submission-guidelines#loc-guidelines-for-specific-study-types) for detailed instructions. **Make sure that all information entered here is included in the Methods section of the manuscript.** | n/a |

| **Format for specific study types**  **Human Subject Research (involving human participants and/or tissue)**   - Give the name of the institutional review board or ethics committee that approved the study - Include the approval number and/or a statement indicating approval of this research - Indicate the form of consent obtained (written/oral) or the reason that consent was not obtained (e.g. the data were analyzed anonymously)   **Animal Research (involving vertebrate animals, embryos or tissues)**   - Provide the name of the Institutional Animal Care and Use Committee (IACUC) or other relevant ethics board that reviewed the study protocol, and indicate whether they approved this research or granted a formal waiver of ethical approval - Include an approval number if one was obtained - If the study involved *non-human primates*, add *additional details* about animal welfare and steps taken to ameliorate suffering - If anesthesia, euthanasia, or any kind of animal sacrifice is part of the study, include briefly which substances and/or methods were applied   **Field Research**  Include the following details if this study involves the collection of plant, animal, or other materials from a natural setting:   - Field permit number - Name of the institution or relevant body that granted permission |  |
| --- | --- |
|  |  |
| **Data Availability**  Authors are required to make all data underlying the findings described fully available, without restriction, and from the time of publication. PLOS allows rare exceptions to address legal and ethical concerns. See the [PLOS Data Policy](http://journals.plos.org/plosone/s/data-availability) and [FAQ](http://journals.plos.org/plosone/s/data-availability#loc-faqs-for-data-policy) for detailed information. | Yes - all data are fully available without restriction |

| A Data Availability Statement describing where the data can be found is required at submission. Your answers to this question constitute the Data Availability Statement and **will be published in the article**, if accepted. |  |
| --- | --- |
| **Important:** Stating ‘data available on request from the author’ is not sufficient. If your data are only available upon request, select ‘No’ for the first question and explain your exceptional situation in the text box. |  |
| Do the authors confirm that all data underlying the findings described in their manuscript are fully available without restriction? |  |
| **Describe where the data may be found in full sentences. If you are copying our sample text, replace any instances of XXX with the appropriate details.**   - If the data are **held or will be held in a public repository**, include URLs, accession numbers or DOIs. If this information will only be available after acceptance, indicate this by ticking the box below. For example: *All XXX files are available from the XXX database (accession number(s) XXX, XXX.*). - If the data are all contained **within the manuscript and/or Supporting Information files**, enter the following: *All relevant data are within the manuscript and its Supporting Information files.* - If neither of these applies but you are able to provide **details of access elsewhere**, with or without limitations, please do so. For example:   *Data cannot be shared publicly because of [XXX]. Data are available from the XXX Institutional Data Access / Ethics Committee (contact via XXX) for researchers who meet the criteria for access to confidential data.*  *The data underlying the results presented in the study are available from (include the name of the third party* | All relevant data are within the manuscript and its Supporting Information files.  Our manuscript is a comparison of diagnostic algorithms in guidelines on nutritional anaemia. To get to the findings and conclusions of our manuscript all relevant guidelines are selected and relevant information selected. All data is in the tables, figures, supplementary files and references. The analyses can be redone by downloading the literature as shown in the reference section. |

| *and contact information or URL).*   - This text is appropriate if the data are owned by a third party and authors do not have permission to share the data.   * typeset |  |
| --- | --- |
| Additional data availability information: |  |

<https://www2.cloud.editorialmanager.com/pone/download.aspx?id=41167409&guid=6fea6a1e-0519-4fb1-a586-d0b3013acc70&scheme=1>Manuscript	[Click here to](https://www2.cloud.editorialmanager.com/pone/download.aspx?id=41167409&guid=6fea6a1e-0519-4fb1-a586-d0b3013acc70&scheme=1)

[access/download;Manuscript;Manuscript_Diagnostic](https://www2.cloud.editorialmanager.com/pone/download.aspx?id=41167409&guid=6fea6a1e-0519-4fb1-a586-d0b3013acc70&scheme=1)

### 1 Comparison of diagnostic algorithms used in guidelines on nutritional

2 **anaemias in adults**

3

4 Margaretha G.M Roemer^1,2^*, Wendy P.J. den Elzen^3,4^* , Rosalinde K.E. Poortvliet^5,6^, Jacobijn
5 Gussekloo^5,6,7^, Steef Kurstjens^8,9^, Bauke A. de Boer^1^, Anne Margreet de Jong^1^ 6
7 1. Atalmedial diagnostic centre, Amsterdam, the Netherlands
8 2. Department of Clinical Chemistry, Haematology & Immunology, Noordwest Ziekenhuisgroep,
9 Alkmaar, the Netherlands
10 3. Laboratory Specialized Diagnostics & Research, Department of Laboratory Medicine,
11 Amsterdam UMC, University of Amsterdam, Amsterdam, the Netherlands
12 4. Amsterdam	Public Health Research Institute & Amsterdam	Gastroenterology and
13 Endocrinology Metabolism, Amsterdam, the Netherlands
14 5. Department of Public Health and Primary Care, Leiden University Medical Center, Leiden, the
15 Netherlands
16 6. LUMC Center for Medicine for Older people, Leiden, the Netherlands
17 7. Department of Internal Medicine, Section of Gerontology and Geriatrics, Leiden University
18 Medical Center, Leiden, the Netherlands
19 8. Laboratory of Clinical Chemistry and Hematology, Jeroen Bosch Hospital, 's Hertogenbosch,
20 the Netherlands
21 9. Laboratory of Clinical Chemistry and Laboratory Medicine, Dicoon BV, location Canisius
22 Wilhelmina Hospital, Nijmegen, the Netherlands
23 * Equal contributions 24
25 **ORCID iD**
26 Margaretha G.M Roemer	0000-0003-0220-3496
27 Wendy P.J. den Elzen	0000-0002-6774-4077
28 Rosalinde K.E. Poortvliet	0000-0001-6521-8770
29 Jacobijn Gussekloo	0000-0001-7186-8278
30 Steef Kurstjens	0009-0008-4409-5079
31 Bauke A. de Boer	0000-0002-4572-1157
32 Anne Margreet de Jong	0000-0002-9638-9115

33

##### 34 Corresponding author

35 Dr. Anne Margreet de Jong
36 Atalmedial Diagnostics Centre, Amsterdam, The Netherlands
37 Louwesweg 6
38 P.O. Box 69641
39 1066 EC Amsterdam
40 The Netherlands
41 Email: [adejong@atalmedial.nl](mailto:adejong@atalmedial.nl)

42

##### 43 Declarations

44 **Conflict of interest:** The authors declare no competing interests.
45 **Funding:** This study had no external funding source.
46 **Ethical approval:** Not applicable.
47 **Author’s contributions:** Conceptualization, study design and data collection: M.G.M.R, W.P.J.d.E.,
48 B.A.d.B. and A.M.d.J.; data preparation and methodology: M.G.M.R, W.P.J.d.E. and A.M.d.J.;
49 investigation: M.G.M.R, W.P.J.d.E. and A.M.d.J.; writing - original draft preparation: M.G.M.R,
50 W.P.J.d.E. and A.M.d.J; writing - review and editing: M.G.M.R, W.P.J.d.E. and A.M.d.J, R.K.E.P., J.G.,
51 S.K., B.A.d.B. All authors have read and agreed to the published version of the manuscript.
52 **Acknowledgements:** not applicable. 53

54	Number of words: 2499 words

55

56	Short title: diagnostic algorithms on anaemia 57

58	Keywords: general practice, anaemia, iron deficiency, ferritin, vitamin B12 deficiency, folate deficiency,

59	guidelines, laboratory aspects

##### 1 Abstract

2

3 *Background*
4 Anaemia is highly prevalent and commonly caused by nutritional deficiencies. Guidelines often
5 include an algorithm to find the underlying cause. Here, we compared diagnostic algorithms and
6 suggested laboratory tests for anaemia in clinical practice in countries with similar healthcare
7 systems, focusing on iron, vitamin B12 and folate deficiency in the general adult population. 8
9 *Methods*
10 We searched for diagnostic guidelines in anaemia in the Trip, Guidelines International Network and country
11 specific databases. To be selected for this systematic review, the guidelines had to include diagnostic
12 criteria or algorithms to determine the cause of anaemia in the general adult population. 13
14 *Results*
15 In total, 14 records were included. For iron deficiency anaemia, guidelines varied in diagnostic criteria
16 ranging from ferritin only, to ferritin in various combinations with a variety of other parameters, with
17 different cut-off values. For vitamin B12 or folate deficiency, besides measurement of vitamin B12 or
18 folate, some guidelines mentioned methylmalonic acid or homocysteine. Quality of evidence underlying
19 cut-offs and parameters was variable, and laboratory aspects were underrepresented. 20
21 *Conclusions*
22 There was great variation in the included diagnostic algorithms, especially for iron deficiency anaemia.
23 Differences in cut-off values were seen, even when using similar diagnostic strategies. Furthermore,
24 supporting evidence was variable. Further research is needed to determine optimal algorithms. Our
25 findings highlight the need for inclusion of relevant laboratory aspects in guidelines, appropriate
26 diagnostics and clinical decision limits. 27

##### 1 Introduction

2

3 Anaemia is a highly prevalent disorder worldwide affecting up to 25% of the population [1, 2], and.
4 is defined as a low number of red blood cells or a low haemoglobin concentration within the red blood
5 cells for the age and sex of the individual [1]. Low haemoglobin concentrations result in a decreased
6 capacity of the blood to carry oxygen to the tissues in the body. Consequently, symptoms include
7 fatigue, weakness, dizziness and shortness of breath. Anaemia is not a diagnosis, but a
8 presentation of an underlying condition. Anaemia is most frequently caused by nutrient deficiencies due
9 to inadequate diets or inadequate absorption, predominantly iron, vitamin B12 and folate.
10 In addition, chronic diseases, infection, kidney failure, genetic disorders, bone marrow
11 pathologies, and acute or chronic haemorrhage can cause anaemia [3, 4]. It is therefore relevant to
12 correctly diagnose the underlying cause of anaemia to adequately treat the patient.
13 General practitioners play a crucial role in the diagnosis and management of anaemia. The
14 diagnosis typically involves a complete blood count (CBC) and further investigative assays
15 to pinpoint the specific aetiology. Premature or unnecessary testing can lead to excessive use of
16 diagnostic blood tests, while failure to test anaemic patients can result in incomplete or missed
17 diagnoses or under- or overtreatment. Both scenarios can potentially increase healthcare costs [5-7].
18 To ensure effective and standardised care, anaemia guidelines have been developed. These
19 guidelines typically encompass initial assessment, diagnostic testing, management and treatment,
20 follow-up and monitoring. For different physiological settings, including age, sex, and pregnancy status
21 or for specific patient groups, different guidelines for diagnosing anaemia have been developed.
22 Different countries have varying requirements for the way in which guidelines are developed, who is
23 involved in their development, and what sources are used. Therefore, the aim of this review
24 was to compare the diagnostic algorithms and suggested laboratory tests for anaemia in clinical practice
25 guidelines in countries with similar healthcare systems for the most common causes of anaemia: iron,
26 vitamin B12 and folate deficiency in adults from the primary care population. This may provide guidance
27 for the development of updated more appropriate guidelines. 28

29

30

31

32

##### 1 Methods

2

3 *Data sources and searches*
4 First, we searched for diagnostic guidelines for anaemia in two guideline databases: Trip database
5 ([https://www.tripdatabase.com](https://www.tripdatabase.com/)) and Guidelines International Network (G-I-N, [http://www.g-i-n.net](http://www.g-i-n.net/)) on

6	January 12th 2022. These databases were selected to search for evidence-based guidelines across the

7 world. We performed an advanced search for ‘anemia’ or ‘anaemia’ in both databases. Secondly, we
8 performed searches in country specific guideline databases (ao. Germany, UK, USA, Canada,
9 Australia) on October 15th 2023. The most recent version of the guidelines was sought and used on

10	February 29th 2024.

11

12 *Guideline selection*
13 To be selected for this systematic review, the guidelines had to include diagnostic criteria or algorithms
14 to find the cause of anaemia in the primary care adult population. We focused on nutritional deficiencies
15 (iron, vitamin B12 and folate). Guidelines had to include criteria for adults and specific guidelines for children
16 were excluded. When multiple guidelines from the same society were found, the most recent version
17 was used. Guidelines focusing on specific populations (e.g. anaemia in pregnancy, chronic kidney
18 disease, dialysis patients, heart failure, cancer), guidelines of which full versions were not available in
19 English, Dutch or German and guidelines of which full versions were inaccessible online or withdrawn,
20 were excluded. Two authors (combinations of MR, WdE, AdJ) independently assessed the titles and
21 full texts of the guidelines retrieved during the searches against the inclusion and exclusion criteria, and
22 selected or rejected as appropriate. Disagreement was resolved by consensus. 23
24 *Data extraction, quality assessment and analysis*
25 The recommendations in the selected guidelines were extracted by two authors (WdE, AdJ) and
26 checked by a third (MR). We rated the quality of the guidelines with a scoring system, based on the
27 evidence underpinning the recommendations. Level 0: no evidence provided, level 1: based on expert
28 opinion, level 2: based on randomised controlled trials / peer reviewed studies before 2010 (for the test
29 of interest), level 3: based on randomised controlled trials / peer reviewed studies 2010 and later (for
30 the test of interest), level 4: based on a systematic review / meta-analysis. Note that guidelines that
31 referred to studies before, as well as after 2010 were evaluated as level 3. 32

##### 33 Results

34 A total of 87 records were identified by the combined searches (Supplementary Table 1). Forty-four
35 records were excluded due to the focus on a specific patient group. The remaining 43 full texts were
36 screened and another 29 were excluded in line with the exclusion criteria (Figure 1).
37 The 14 included guidelines originated from 6 different countries (US, UK, Germany, Canada, Qatar and the
38 Netherlands).

39

40 *Patient population*
41 We focused on guidelines in the primary care patient population. Nonetheless, a plethora of different
42 anaemia guidelines were found that included specific organs, patient groups and underlying diseases.
1 Therefore, we decided to select all guidelines focusing on the “general population”. This included
2 primary care, as well as secondary care guidelines in otherwise healthy people.
3 Age definitions for patient populations that were included in the different guidelines were
4 variable. Most of the guidelines did not state a specific age definition [8-15], while others focused on
5 age ≥5 years [16], >12 years [17], ≥16 years [18, 19], ≥18 years [20] or on older adults [21]. Although
6 all guidelines acknowledged that older people have a higher risk of developing anaemia, and/or
7 suggested further diagnostics, no age-specific diagnostic algorithms were provided. 8
9 *Definition of anaemia*
10 According to the World Health Organization (WHO), anaemia is defined as a haemoglobin level <12.0
11 g/dL in women and <13.0 g/dL in men [1]. Most of the guidelines adhered to this definition [8, 9, 11-14,
12 17, 19, 20], although one guideline used <13.5 g/dL in men [16] and one guideline used <11.5 g/dL in
13 women (Table 1) [18]. Three guidelines did not specify a value for low haemoglobin, but referred to
14 local laboratory reference values [10, 15, 21].

15

##### 16 Table 1. Summarized haemoglobin cut-offs in the included guidelines to define anaemia

| **Guideline** | **Year** | **Country** | **Haemoglobin level** | **Evidence** | **Ref** |
| --- | --- | --- | --- | --- | --- |
| NHS Greater Glasgow and Clyde | 2022 | UK | M <13 g/dL,  F <11.5 g/dL | 1 | 18 |
| Centre for Perioperative Care | 2022 | UK | M <13 g/dL, F <12 g/dL * | 4 | 8 |
| Association of Scientific Medical Societies Germany | 2021 | Germany | M <13 g/dL,  F <12 g/dL | 4 | 9 |
| British Society of Gastroenterology | 2021 | UK | < N | NA | 10 |
| National Institute for Health and Care Excellence | 2021 | UK | M <13 g/dL,  F <12 g/dL | 4 | 17 |
| Dutch College of General Practitioners | 2021 | The Netherlands | M < 13 g/dL, F <12 g/dL # | 4 | 11 |
| Ministry of Public Health Qatar | 2020 | Qatar | M <13 g/dL,  F <12 g/dL | 4 | 12 |
| American Gastroenterological Association | 2020 | USA | M <13 g/dL,  F <12 g/dL | 4 | 13 |
| National Institute for Health and Care Excellence | 2020 | UK | M <13 g/dL, F <12 g/dL # | 4 | 19 |
| Royal College of Nursing | 2019 | UK | M <13 g/dL,  F <12 g/dL | 4 | 14 |
| BC guidelines | 2019 | Canada | < N | NA | 15 |
| Association of Scientific Medical Societies Germany | 2018 | Germany | M <13 g/dL,  F <12 g/dL, Drop > 2 g/dL | 4 | 20 |
| Toward Optimized Practice | 2018 | Canada | M <13.5 g/dL,  F <12 g/dL # | 1 | 16 |
| American Medical Directors Association | 2007 | USA | < N | NA | 21 |

17 Abbreviations: Ref, reference; M, male; F, female; N, normal; NA, not applicable
18 * international consensus statement in the peri-operative period: Hb <13 g/dL for both sexes [22]
19 # Suggested cut-off values for assessment

20

21

1 *Iron deficiency anaemia*
2 For iron deficiency anaemia, there was a lot of variation in the included diagnostic criteria, as
3 summarised in Table 2. Diagnostic criteria ranged from only ferritin, to ferritin in various combinations
4 with mean corpuscular volume (MCV), iron, transferrin, transferrin saturation, total iron binding capacity
5 (TIBC), C-reactive protein (CRP), erythrocyte sedimentation rate (ESR), reticulocyte
6 haemoglobin content (Ret-Hb) and reticulocytes count with different cut-off values. Of note, even when a similar
7 strategy was used, differences in cut-off values were seen.
8 Testing of MCV was recommended in seven guidelines, in combination with ferritin [9-12, 16,
9 20, 21]. Other erythrocyte indices (mean corpuscular haemoglobin, mean corpuscular haemoglobin
10 concentration, red cell distribution width) were variably mentioned in a minority of the guidelines, and
11 therefore not included in Table 2.
12 All guidelines included ferritin for the diagnosis of iron deficiency anaemia. Cut-offs ranged from
13 <15 µg/L [15, 18] to <30 µg/L [8, 14, 17, 20] and <45 µg/L [13] or referred to the local lower limit of reference
14 range [10-12, 21]. Two guidelines used different ferritin cut-offs for men and women [9, 16].
15 The fact that ferritin is an acute phase protein and thus increases with infection or inflammation
16 was recognized in all guidelines. Nonetheless, some guidelines only mentioned it [9, 12, 13, 17, 21],
17 while other guidelines specified the ferritin level in inflammation below which iron deficiency could still be considered [11,
18 15, 16, 18, 20] and/or recommended measurement of CRP/ESR to confirm the presence of
19 inflammation [8, 10, 14]. Additional testing of transferrin saturation when a normal ferritin level was
20 found was recommended in five guidelines [8, 10, 14, 20, 21].
21 Testing of the serum iron was recommended in three guidelines, in combination with MCV, ferritin,
22 transferrin, transferrin saturation and/or TIBC [11, 12, 21]. Lastly, three guidelines recommended
23 examination of Ret-Hb or reticulocytes [8-10]. 24
25 *Vitamin B12 or folate deficiency anaemia*
26 Six vitamin B12 and folate deficiency anaemia guidelines were included. Most of these guidelines
27 contained a combined anaemia diagnostic algorithm with iron deficiency [8, 11, 12, 20, 21] and one
28 guideline specifically focused on vitamin B12 and folate deficiency (Table 3) [19]. All guidelines
29 mentioned that vitamin B12 and/or folate anaemia is macrocytic. Four guidelines stated that the
30 anaemia may also be normocytic [8, 11, 19, 21].
31 For vitamin B12, one guideline recommended a specific cut-off [19], the other guidelines
32 referred to local laboratory reference values. With regards to the use of total vitamin B12 (cobalamin)
33 or active vitamin B12 (holotranscobalamin), four guidelines specifically recommended the (initial) testing
34 of total vitamin B12 [8, 11, 19, 20].
35 In addition to vitamin B12, four guidelines mentioned testing for methylmalonic acid (MMA)
36 and/or homocysteine, always in addition to vitamin B12 [12, 21] or when the vitamin B12 level is in the
37 lower normal reference range [11, 20].
38 For folate, most guidelines recommended testing for serum folate only [8, 11, 21]. Two guidelines
39 included RBC folate as a diagnostic criterion [19, 20]. One guideline recommended lactate
40 dehydrogenase (LDH) and reticulocytes for folate, as well as vitamin B12 deficiency [11].

##### 1 Table 2. Summarized diagnostic algorithms of the included iron deficiency anaemia guidelines.

| **Guideline** | **Year** | **Country** | **MCV**  **(fl)** | **Ferritin (µg/L)** | **Iron** | **Transferrin** | **Transferrin sat (%)** | **TIBC** | **CRP (mg/L) or ESR** | **Ret-He / Reticulocytes** | **Evidence** | **Ref** |
| --- | --- | --- | --- | --- | --- | --- | --- | --- | --- | --- | --- | --- |
| NHS Greater Glasgow and Clyde | 2022 | UK |  | < 15 or 15 - 50 † |  |  |  |  |  |  | 1 | 18 |
| Centre for Perioperative Care | 2022 | UK |  | < 30 * or 30 - 100 † |  |  | <20% * |  | > 5 † | < 30 pg * | 3 | 8 |
| Association of Scientific Medical Societies Germany | 2021 | Germany | < 78 | M < 35, F < 23  or ≥ N † |  |  |  |  |  | < 28 pg / ≤ N | 2 | 9 |
| British Society of Gastroenterology | 2021 | UK | < N* | < N or N * |  |  | Low * |  | > N * | < N * | 4 | 10 |
| National Institute for Health and Care Excellence | 2021 | UK |  | < 30 ‡ |  |  |  |  |  |  | 3 | 17 |
| Dutch College of General Practitioners | 2021 | the Netherlands | ≤ N | < N  or < 100 † * | < N * | > N * |  |  |  |  | 2 | 11 |
| Ministry of Public Health Qatar | 2020 | Qatar | < N | ≤ N ‡ | < N |  |  | ≥ N |  |  | 3 | 12 |
| American Gastroenterological Association | 2020 | USA |  | < 45 ‡ |  |  |  |  |  |  | 4 | 13 |
| Royal College of Nursing | 2019 | UK |  | < 30 or 30 - 100 ^ |  |  | < 20% ^ |  | > 5 ^ |  | 1 | 14 |
| BC guidelines | 2019 | Canada |  | < 15 or 15 - 30 $  or < 100 † |  |  |  |  |  |  | 2 | 15 |
| Association of Scientific Medical Societies Germany | 2018 | Germany | < N | < 30 * or 30 - 100 * or > 300 †# |  |  | < 20% * or  †# |  |  |  | 3 | 20 |
| Toward Optimized Practice | 2018 | Canada | < 75 * | M < 30 *, F < 13  or < 100 † |  |  |  |  |  |  | 3 | 16 |
| American Medical Directors Association | 2007 | USA | < N | < N or < N #  or N † | < N # † | > N # | < N # † | > N # |  |  | 2 | 21 |

2 Abbreviations: MCV, mean corpuscular volume; sat, saturation; TIBC, total iron binding capacity; CRP, C-reactive protein; ESR, erythrocyte sedimentation rate; Ret-He, reticulocyte haemoglobin
3 equivalent; Ref, reference; M, male; F, female; N, normal; pg, picograms.
4 * and/or
5 # and
6 ^ or
7 † Patients with chronic (inflammatory) disease, active inflammation or malignancy
8 ‡ Ferritin is an acute phase protein and increases in patients with infection or inflammation
9 $ Probably iron deficiency

##### 1 Table 3. Summarized diagnostic algorithms which included vitamin B12 / folate deficiency anaemia guidelines.

|  | | | | **Vitamin B12** | | | | **Folate** | | | | **Folate and vit. B12** | |  | |
| --- | --- | --- | --- | --- | --- | --- | --- | --- | --- | --- | --- | --- | --- | --- | --- |
| **Guideline** | **Year** | **Country** | **MCV**  **(fl)** | **Vit. B12**  **(pmol/L)** | **Active vit.**  **B12** | **MMA** | **Hcy** | **Folate (nmol/L)** | **RBC**  **folate (nmol/L)** | **MMA** | **Hcy** | **LDH** | **Reticulocytes** | **Evidence** | **Ref** |
| Centre for Perioperative Care | 2022 | UK | ≥ N | < N |  |  |  | < N |  |  |  |  |  | 3 | 8 |
| Dutch College of General Practitioners | 2021 | the Netherlands | ≥ N | < N or low N * |  | Optional (1st) * | Optional (2nd) * | < N |  |  |  | > N | < N | 3 | 11 |
| National Institute for Health and Care Excellence | 2020 | UK | ≥ N | < 148 |  |  |  | < 7 or N # | < 340 # |  |  |  |  | 3 | 19 |
| Ministry of Public Health Qatar | 2020 | Qatar | > N | < N |  | > N |  | < N |  | N |  |  |  | 3 | 12 |
| Association of Scientific Medical Societies Germany | 2018 | Germany | > N | < N or low N * | Optional * | Optional * | Optional * | < N ^ | Confirm |  | Optional |  |  | 3 | 20 |
| American Medical Directors Association | 2007 | USA | ≥ N | < N |  | > N | > N | < N |  |  |  |  |  | 2 | 21 |

2 Abbreviations: MCV, mean corpuscular volume; Vit, vitamin; MMA, methylmalonic acid; Hcy, homocysteine; RBC, red blood cell; LDH, lactate dehydrogenase; Ref, reference; N, normal
3 * and / or
4 # with normal vit. B12
5 ^ related to food intake, a second fasting blood test is required
1 *Quality of the evidence*
2 The quality of the included guidelines was evaluated and ranked for the indicated parameters (colour-
3 coded in Table 1, 2 and 3). To define anaemia, WHO haemoglobin cut-offs were used in most guidelines
4 [8, 9, 11-14, 17, 19-21]. Given the systematic nature of the evidence and recently unchanged cut-offs
5 [23], this was regarded as level 4. For iron deficiency anaemia, two guidelines were considered level 4
6 [10, 13], five guidelines level 3 [8, 12, 16, 17, 20], four guidelines level 2 [9, 11, 15, 21] and two
7 guidelines level 1 [14, 18]. It must be noted that guidelines frequently referred to each other, but
8 nonetheless used variable cut-offs. Five out of six vitamin B12 and folate deficiency anaemia guidelines
9 were considered level 3 [8, 11, 12, 19, 20] and one guideline level 2 [21].

10

11 *Laboratory aspects*
12 Laboratory aspects were only scarcely reported in the included guidelines. Even though laboratory
13 measurements are used in all guidelines, none of the guidelines specified which laboratory kit manufacturer or
14 measurement method was used to determine the specific cut-off values, and the type of material (e.g.
15 plasma or serum) that should be used for the test of interest. 16

17

##### 1 Discussion

2 In primary care, the CBC is one of the most common blood tests, often done as a routine check-up.
3 When a low haemoglobin is found, follow-up tests are used to investigate the cause of anaemia [24].
4 This systematic review elucidated similarities and differences in diagnostic algorithms in evidence-
5 based guidelines for the most common causes of anaemia: iron, vitamin B12 and folate deficiency. An
6 overview of our observations and recommendations for future guidelines is summarised in Figure 2.
7 In brief, we observed that primary and secondary care guidelines are intertwined, and that there are
8 insufficient attention to diverse aspects. Furthermore, a lot of variation was seen in the recommended
9 diagnostic criteria, cut-off values and evidence underpinning the algorithms, even in the presence of
10 similar diagnostic strategies. Importantly, even though laboratory testing is proposed in all guidelines,
11 the specifics of these tests are not described. 12
13 *Patient population and diversity in diagnostic algorithms*
14 Primary care, as well as secondary care guidelines for otherwise healthy people were included. For
15 future guidelines, a focus on either the primary or secondary care setting is endorsed, as well as
16 alignment between the recommendations of primary care and secondary care. Similar misalignments
17 were observed in the field of subclinical hyperthyroidism [25].
18 Remarkably, no distinctions were made by sex or age in most of the diagnostic
19 algorithms. However, research shows that sex differences may be of importance [26]. Nonetheless, the
20 WHO did not find enough evidence for a change of ferritin cut-off values by sex [27]. Additionally, the
21 diagnostic work-up and treatment of anaemia in older patients may be different from other age groups
22 [28-30]. Hence, we recommend including at least age and sex, in the diagnostic algorithms. 23
24 *Included parameters in diagnostic algorithms*
25 After anaemia is identified, most of the guidelines used the MCV to define the cause of the anaemia.
26 However, a normal MCV does not exclude the presence of iron, vitamin B12 or folate deficiency
27 [31]. Therefore, MCV should be mainly used as a supporting parameter.
28 For iron deficiency anaemia, ferritin was included in all algorithms, however, the cut-offs
29 were variable, as has been previously described [32-35] and no proper justification for these cut-offs
30 was provided. Despite an extensive review performed by the WHO, insufficient evidence was found for
31 a consensus on the ferritin threshold and therefore the cut-off of <15 µg/L remained unchanged [27, 32,
32 33]. In the presence of inflammation, WHO recommends a cut-off of 70 µg/L to diagnose iron deficiency
33 anaemia [27]. However, most guidelines gave a ferritin cut-off of 100 µg/L in this situation. There
34 is no consensus about which additional parameters should be evaluated. Furthermore, ferritin levels
35 are affected by recent oral iron supplementation intake, coffee and other dietary factors[36]. Therefore,
36 it is recommended to fast overnight and withhold supplements before measuring ferritin[37]. These
37 factors are not adequately emphasized in guidelines, which can lead to diagnostic confusion.
38 For vitamin B12 and folate deficiency, most guidelines referred to local lower limit of reference
39 values. Given the variation of used cut-offs in literature [38, 39], this is a sensible approach. Not all
40 guidelines specify if total vitamin B12 or active vitamin B12 should be tested. Also, additional testing
1 of MMA and homocysteine is not consistently advised. To date, the most reliable parameter to detect
2 vitamin B12 deficiency is still a matter of debate. According to Herbert’s hypothesis from the 1980’s, active vitamin B12
3 is an earlier and more sensitive indicator of vitamin B12 deficiency [40, 41], which was supported by
4 later studies [42-45]. Nonetheless, other studies contradict this and state that the reliability of active and
5 total vitamin B12 is similar [46, 47].
6 These findings highlight the need for additional research to determine the optimal
7 parameters and cut-offs to diagnose anaemia caused by nutritional deficiencies. 8

9	*Laboratory aspects in guidelines*

10 There is limited awareness of the laboratory aspects in anaemia guidelines, although all the algorithms use
11 laboratory tests to determine the cause of anaemia. It must be noted that the ferritin, vitamin B12 and
12 folate assays are not harmonised between platforms and there are substantial inter-analyser
13 differences. The lack of harmonisation may result in different values when the same sample is
14 measured in different labs using different analyser platforms, and thus reference ranges may differ between laboratories [48-52]. Concentrations may also be
15 dependent on the type of material, e.g. serum or plasma that is used for the analysis. In general, serum
16 as well as plasma is acceptable for analysis. Nonetheless, higher or lower values may be found in
17 specific types of material for ferritin, vitamin B12 and folate [44, 51, 53]. Therefore, if cut-offs are
18 mentioned, the used sample type, analyser platform and method should be specified. Applying
19 inappropriate cut-offs can lead to misinterpretation and may cause under- or overdiagnosis..
20 Given the lack of harmonisation of ferritin, vitamin B12 and folate, at this point we recommend referring
21 to local laboratory reference values or cut-offs provided by the local laboratory. 22
23 *Strengths and limitations*
24 To our knowledge, this is the first study that systematically reviewed anaemia guidelines focusing on
25 the diagnostics of the most common causes of anaemia. In addition to summarising diagnostic
26 strategies, we present recommendations that may be used for developing new guidelines. A limitation
27 of this study is that we only selected guidelines of countries with comparable healthcare systems that
28 were available in Dutch, German or English, potentially missing other relevant guidelines. Nonetheless,
29 our review provides an overview of diagnostic strategies ofguidelines from all over the world. 30
31 *Conclusion*
32 There was considerable variation in the included diagnostic criteria for anaemia caused by nutritional
33 deficiencies in guidelines for the adult population. Even when similar diagnostic strategies were used,
34 differences in cut-off values were seen and the supporting evidence was variable. Further research is
35 needed to determine optimal algorithms. Even though laboratory tests are a fundamental aspect of the
36 diagnostic algorithms, description of relevant laboratory aspects was lacking. For future guidelines, it
37 will be important to include relevant laboratory aspects in guidelines, considering appropriate
38 diagnostics and clinical decision limits. 39

40

##### 41 Supporting information

1

2	Supplementary Table 1. Exclusion table

3

4	Supplementary Table 2. PRISMA abstract checklist 5

6	Supplementary Table 3. PRISMA checklist

7

##### 1 References

2 1.	World Health Organization. Worldwide prevalence of anaemia 1993-2005 - WHO global
3 database on anaemia. Edited by Bruno de Benoist, Erin McLean, Ines Egli and Mary Cogswell 2008.
4 2.	Safiri S, Kolahi AA, Noori M, Nejadghaderi SA, Karamzad N, Bragazzi NL, et al. Burden of
5 anemia and its underlying causes in 204 countries and territories, 1990-2019: results from the Global
6 Burden of Disease Study 2019. J Hematol Oncol. 2021;14(1):185.
7 3.	Cascio MJ, DeLoughery TG. Anemia: Evaluation and Diagnostic Tests. Med Clin North Am.
8 2017;101(2):263-84.
9 4.	Kassebaum NJ, Collaborators GBDA. The Global Burden of Anemia. Hematol Oncol Clin North
10 Am. 2016;30(2):247-308.
11 5.	de Jong AM, Bozic C, Candido F, van Stralen KJ, de Boer BA. [The cause of anemia is
12 underdiagnosed in primary care]. Ned Tijdschr Geneeskd. 2023;167.
13 6.	Zhi M, Ding EL, Theisen-Toupal J, Whelan J, Arnaout R. The landscape of inappropriate
14 laboratory testing: a 15-year meta-analysis. PLoS One. 2013;8(11):e78962.
15 7.	Kip MMA, Oonk MLJ, Levin MD, Schop A, Bindels PJE, Kusters R, et al. Preventing overuse of
16 laboratory diagnostics: a case study into diagnosing anaemia in Dutch general practice. BMC Med
17 Inform Decis Mak. 2020;20(1):178.
18 8.	Centre for Perioperative Care. Guideline for the Management of Anaemia in the Perioperative
19 Pathway. Available from [https://cpoc.org.uk/guidelines-resources-guidelines/anaemia-perioperative-](https://cpoc.org.uk/guidelines-resources-guidelines/anaemia-perioperative-pathway)
20 [pathway.](https://cpoc.org.uk/guidelines-resources-guidelines/anaemia-perioperative-pathway) 2022.
21 9.	Behnisch W, Muckenthaler M, Kulozik A. Arbeitsgemeinschaft der Wissenschaftlichen
22 Medizinischen	Fachgesellschaften.Eisenmangelanamie.	Available	from
23 [https://register.awmf.org/de/leitlinien/detail/025-021.](https://register.awmf.org/de/leitlinien/detail/025-021) 2021.
24 10.	Snook J, Bhala N, Beales ILP, Cannings D, Kightley C, Logan RPH, et al. British Society of
25 Gastroenterology. Guidelines for the Management of Iron Deficiency Anaemia in Adults. Gut.
26 2021;70:2030-51.
27 11.	Bouma M, Burgers JS, Drost B, Den Elzen WPJ, Luchtman T, Oosterhuis WP, et al. Nederlands
28 Huisarts	Genootschap	(NHG)-Standaard	Anemie	(M76).	Available	from
29 [https://richtlijnen.nhg.org/standaarden/anemie.](https://richtlijnen.nhg.org/standaarden/anemie) 2021.
30 12.	Ministry of Public Health Qatar. National Clinical Guidelines: The Assessment & Management
31 of Common Anaemias in Adults. Available from [https://www.isbtweb.org/resource/the-assessment-and-](https://www.isbtweb.org/resource/the-assessment-and-management-of-common-anaemias-in-adults--1--pdf.html)
32 [management-of-common-anaemias-in-adults--1--pdf.html.](https://www.isbtweb.org/resource/the-assessment-and-management-of-common-anaemias-in-adults--1--pdf.html) 2020.
33 13.	Ko CW, Siddique SM, Patel A, Harris A, Sultan S, Altayar O, et al. American
34 Gastroenterological Association. AGA Clinical Practice Guidelines on the Gastrointestinal Evaluation of
35 Iron Deficiency Anemia. Gastroenterology. 2020;159:1085-94.
36 14.	Royal	College	of	Nursing.	Iron	deficiency	anaemia	in	adults.	Available	from
37 [https://www.rcn.org.uk/Professional-Development/publications/pub-007460.](https://www.rcn.org.uk/Professional-Development/publications/pub-007460) 2019.
38 15.	Ministry of Health, British Columbia. Doctors of BC. BCGuidelines. Iron Deficiency - Diagnosis
39 and Management. Available from [https://www2.gov.bc.ca/gov/content/health/practitioner-professional-](https://www2.gov.bc.ca/gov/content/health/practitioner-professional-resources/bc-guidelines/iron-deficiency)
40 [resources/bc-guidelines/iron-deficiency.](https://www2.gov.bc.ca/gov/content/health/practitioner-professional-resources/bc-guidelines/iron-deficiency) 2019.
41 16.	Toward Optimized Practice Iron Defiency Anemia Committee. Iron deficiency anemia clinical
42 practice	guideline.	Edmonton,	AB:	Toward	Optimized	Practice.	Available	from
43 <https://www.albertadoctors.org/media/atabokv2/iron-deficiency-anemia-guideline.pdf>. 2018.
44 17.	National Institute for Care and health Excellence (NICE). Iron deficiency anaemia. Available
45 from cks.nice.org.uk 2021.
46 18.	NHS Greater Glasgow and Clyde. Clinical guideline. Iron deficiency Anaemia in Adults: Oral
47 and	Intravenous	Iron	Therapy	Treatment.	Available	from
48 [https://rightdecisions.scot.nhs.uk/m/2295/ida.pdf.](https://rightdecisions.scot.nhs.uk/m/2295/ida.pdf) 2022.
49 19.	National Institute for Care and health Excellence (NICE). Anaemia - B12 and folate deficiency.
50 Available from cks.nice.org.uk. 2020.
51 20.	Deutsche Gesellschaft fur Anasthesiologie und Intensivmedizin in Kooperation mit der
52 Arbeitsgemeinschaft der Wissenschaftlichen Medizinischen Fachgesellschaften. Praoperative Anamie:
53 Diagnostik	und	Therapie	der	Praoperative	Anamie.	Available	from
54 [https://register.awmf.org/de/leitlinien/detail/001-024.](https://register.awmf.org/de/leitlinien/detail/001-024) 2018.
55 21.	American Medical Directors Association. Anemia in the long-term care setting. Available from
56 [https://paltc.org/products/anemia-long-term-care-setting.](https://paltc.org/products/anemia-long-term-care-setting) 2007.
57 22.	Munoz M, Acheson AG, Auerbach M, Besser M, Habler O, Kehlet H, et al. International
58 consensus statement on the peri-operative management of anaemia and iron deficiency. Anaesthesia.
59 2017;72(2):233-47.
1 23.	World Health Organization. Guideline on haemoglobin cutoffs to define anaemia in individuals
2 and populations. 2024.
3 24.	Fanshawe TR, Ordonez-Mena JM, Turner PJ, Bruel AVD, Shine B, Hayward GN. Frequencies
4 and patterns of laboratory test requests from general practice: a service evaluation to inform point-of-
5 care testing. J Clin Pathol. 2018;71(12):1065-71.
6 25.	Ursem SR, Boelen A, Bruinstroop E, Elders PJM, Gussekloo J, Poortvliet RKE, et al. A
7 systematic review of subclinical hyperthyroidism guidelines: a remarkable range of recommendations.
8 Eur Thyroid J. 2024;13(3).
9 26.	Levi M, Simonetti M, Marconi E, Brignoli O, Cancian M, Masotti A, et al. Gender differences in
10 determinants of iron-deficiency anemia: a population-based study conducted in four European
11 countries. Ann Hematol. 2019;98(7):1573-82.
12 27.	World Health Organization. WHO guideline on use of ferritin concentrations to assess iron
13 status in individuals and populations. 2020.
14 28.	den Elzen WP, Westendorp RG, Frolich M, de Ruijter W, Assendelft WJ, Gussekloo J. Vitamin
15 B12 and folate and the risk of anemia in old age: the Leiden 85-Plus Study. Arch Intern Med.
16 2008;168(20):2238-44.
17 29.	den Elzen WP, Gussekloo J. Anaemia in older persons. Neth J Med. 2011;69(6):260-7.
18 30.	Smelt AF, Gussekloo J, Bermingham LW, Allen E, Dangour AD, Eussen SJ, et al. The effect of
19 vitamin B12 and folic acid supplementation on routine haematological parameters in older people: an
20 individual participant data meta-analysis. Eur J Clin Nutr. 2018;72(6):785-95.
21 31.	Schop A, Stouten K, Riedl JA, van Houten RJ, Leening MJG, Bindels PJE, et al. The accuracy
22 of mean corpuscular volume guided anaemia classification in primary care. Fam Pract. 2021;38(6):735-
23 9.
24 32.	Garcia-Casal MN, Pasricha SR, Martinez RX, Lopez-Perez L, Pena-Rosas JP. Are Current
25 Serum and Plasma Ferritin Cut-offs for Iron Deficiency and Overload Accurate and Reflecting Iron
26 Status? A Systematic Review. Arch Med Res. 2018;49(6):405-17.
27 33.	Garcia-Casal MN, Pasricha SR, Martinez RX, Lopez-Perez L, Pena-Rosas JP. Serum or
28 plasma ferritin concentration as an index of iron deficiency and overload. Cochrane Database Syst Rev.
29 2021;5(5):CD011817.
30 34.	Peyrin-Biroulet L, Williet N, Cacoub P. Guidelines on the diagnosis and treatment of iron
31 deficiency across indications: a systematic review. Am J Clin Nutr. 2015;102(6):1585-94.
32 35.	Richardson D, Brown K, Rusch J, Verburgh E, Louw V, Opie J. From the marrow to the blood:
33 optimising the diagnosis of iron deficiency in the setting of inflammation. Pathology. 2025;57(1):87-93.
34 36.	von Siebenthal HK, Moretti D, Zimmermann MB, Stoffel NU. Effect of dietary factors and time
35 of day on iron absorption from oral iron supplements in iron deficient women. Am J Hematol.
36 2023;98(9):1356-63.
37 37.	Balendran S, Forsyth C. Non-anaemic iron deficiency. Aust Prescr. 2021;44(6):193-6.
38 38.	Raghavan R, Ashour FS, Bailey R. A Review of Cutoffs for Nutritional Biomarkers. Adv Nutr.
39 2016;7(1):112-20.
40 39.	Aparicio-Ugarriza R, Palacios G, Alder M, Gonzalez-Gross M. A review of the cut-off points for
41 the diagnosis of vitamin B12 deficiency in the general population. Clin Chem Lab Med. 2015;53(8):1149-
42 59.
43 40.	Herbert V. The 1986 Herman award lecture. Nutrition science as a continually unfolding story:
44 the folate and vitamin B-12 paradigm. Am J Clin Nutr. 1987;46(3):387-402.
45 41.	Herbert V. Staging vitamin B-12 (cobalamin) status in vegetarians. Am J Clin Nutr. 1994;59(5
46 Suppl):1213S-22S.
47 42.	Heil SG, de Jonge R, de Rotte MC, van Wijnen M, Heiner-Fokkema RM, Kobold AC, et al.
48 Screening for metabolic vitamin B12 deficiency by holotranscobalamin in patients suspected of vitamin
49 B12 deficiency: a multicentre study. Ann Clin Biochem. 2012;49(Pt 2):184-9.
50 43.	Jarquin Campos A, Risch L, Nydegger U, Wiesner J, Vazquez Van Dyck M, Renz H, et al.
51 Diagnostic Accuracy of Holotranscobalamin, Vitamin B12, Methylmalonic Acid, and Homocysteine in
52 Detecting B12 Deficiency in a Large, Mixed Patient Population. Dis Markers. 2020;2020:7468506.
53 44.	Nexo E, Hoffmann-Lucke E. Holotranscobalamin, a marker of vitamin B-12 status: analytical
54 aspects and clinical utility. Am J Clin Nutr. 2011;94(1):359S-65S.
55 45.	Valente E, Scott JM, Ueland PM, Cunningham C, Casey M, Molloy AM. Diagnostic accuracy of
56 holotranscobalamin, methylmalonic acid, serum cobalamin, and other indicators of tissue vitamin
57 B(1)(2) status in the elderly. Clin Chem. 2011;57(6):856-63.
58 46.	Golding PH. Holotranscobalamin (HoloTC, Active-B12) and Herbert's model for the
59 development of vitamin B12 deficiency: a review and alternative hypothesis. Springerplus.
60 2016;5(1):668.
1 47.	Remacha AF, Sarda MP, Canals C, Queralto JM, Zapico E, Remacha J, et al. Role of serum
2 holotranscobalamin (holoTC) in the diagnosis of patients with low serum cobalamin. Comparison with
3 methylmalonic acid and homocysteine. Ann Hematol. 2014;93(4):565-9.
4 48.	Hoofnagle AN. Harmonization of blood-based indicators of iron status: making the hard work
5 matter. Am J Clin Nutr. 2017;106(Suppl 6):1615S-9S.
6 49.	Devalia V, Hamilton MS, Molloy AM, British Committee for Standards in H. Guidelines for the
7 diagnosis and treatment of cobalamin and folate disorders. Br J Haematol. 2014;166(4):496-513.
8 50.	Choy KW, Sezgin G, Wijeratne N, Calleja J, Liwayan R, Rathnayake G, et al. Assessment of
9 analytical bias in ferritin assays and impact on functional reference limits. Pathology. 2022;54(3):302-
10 7.
11 51.	Owen WE, Roberts WL. Comparison of five automated serum and whole blood folate assays.
12 Am J Clin Pathol. 2003;120(1):121-6.
13 52.	Kurstjens S, van Dam AD, Oortwijn E, den Elzen WPJ, Candido F, Kusters R, et al.
14 Inconsistency in ferritin reference intervals across laboratories: a major concern for clinical decision
15 making. Clin Chem Lab Med. 2025;63(3):600-10.
16 53.	Ji Y, Faddy H, Hyland C, Flower R. A plasma ferritin is not always a serum ferritin. Pathology.
17 2015;47:S89-S90.

18

Figure 1	[Click here to access/download;Figure;Fig 1.tif](https://www2.cloud.editorialmanager.com/pone/download.aspx?id=41167337&guid=da6615bb-3b00-4161-8a74-94fbbe710af8&scheme=1)[<https://www2.cloud.editorialmanager.com/pone/download.aspx?id=41167337&guid=da6615bb-3b00-4161-8a74-94fbbe710af8&scheme=1>](https://www2.cloud.editorialmanager.com/pone/download.aspx?id=41167337&guid=da6615bb-3b00-4161-8a74-94fbbe710af8&scheme=1)

Figure 2	[Click here to access/download;Figure;Fig 2.tif](https://www2.cloud.editorialmanager.com/pone/download.aspx?id=41167339&guid=635506c8-a439-410c-aae0-d287c6db4e54&scheme=1)[<https://www2.cloud.editorialmanager.com/pone/download.aspx?id=41167339&guid=635506c8-a439-410c-aae0-d287c6db4e54&scheme=1>](https://www2.cloud.editorialmanager.com/pone/download.aspx?id=41167339&guid=635506c8-a439-410c-aae0-d287c6db4e54&scheme=1)

PRISMA abstract_checklist

[Click here to access/download](https://www2.cloud.editorialmanager.com/pone/download.aspx?id=41177869&guid=da836ffa-bfeb-45c9-93f1-2657cfa44e4b&scheme=1)

# [Supporting Information](https://www2.cloud.editorialmanager.com/pone/download.aspx?id=41177869&guid=da836ffa-bfeb-45c9-93f1-2657cfa44e4b&scheme=1)

## [2025 PRISMA_2020_abstract_checklist.docx](https://www2.cloud.editorialmanager.com/pone/download.aspx?id=41177869&guid=da836ffa-bfeb-45c9-93f1-2657cfa44e4b&scheme=1)

PRISMA checklist

[Click here to access/download](https://www2.cloud.editorialmanager.com/pone/download.aspx?id=41177870&guid=cdeff1f8-2eb4-4012-89cf-013ea4bf14e9&scheme=1)

# [Supporting Information](https://www2.cloud.editorialmanager.com/pone/download.aspx?id=41177870&guid=cdeff1f8-2eb4-4012-89cf-013ea4bf14e9&scheme=1)

## [2025 PRISMA_2020_checklist.docx](https://www2.cloud.editorialmanager.com/pone/download.aspx?id=41177870&guid=cdeff1f8-2eb4-4012-89cf-013ea4bf14e9&scheme=1)

Exclusion table

[Click here to access/download](https://www2.cloud.editorialmanager.com/pone/download.aspx?id=41167477&guid=31b45873-c567-4a20-a590-8219ac81ae46&scheme=1)

[**Supporting Information** Supplementary Table 1. Exclusion table.xlsx](https://www2.cloud.editorialmanager.com/pone/download.aspx?id=41167477&guid=31b45873-c567-4a20-a590-8219ac81ae46&scheme=1)

Revised Manuscript with Track Changes

1	**Comparison of d~~D~~iagnostic algorithms in guidelines on nutritional**

2	**anaemias in adults~~: a systematic review~~**

3

4 Margaretha G.M Roemer^1,2^*, Wendy P.J. den Elzen^3,4^* , Rosalinde K.E. Poortvliet^5,6^, Jacobijn
5 Gussekloo^5,6,7^, Steef Kurstjens^8,9^, Bauke A. de Boer^1^, Anne Margreet de Jong^1^ 6
7 1. Atalmedial diagnostic centre, Amsterdam, the Netherlands
8 2. Department of Clinical Chemistry, Haematology & Immunology, Noordwest Ziekenhuisgroep,
9 Alkmaar, the Netherlands
10 3. Laboratory Specialized Diagnostics & Research, Department of Laboratory Medicine,
11 Amsterdam UMC, University of Amsterdam, Amsterdam, the Netherlands
12 4. Amsterdam Public Health Research Institute & Amsterdam Gastroenterology and
13 Endocrinology Metabolism, Amsterdam, the Netherlands
14 5. Department of Public Health and Primary Care, Leiden University Medical Center, Leiden, the
15 Netherlands
16 6. LUMC Center for Medicine for Older people, Leiden, the Netherlands
17 7. Department of Internal Medicine, Section of Gerontology and Geriatrics, Leiden University
18 Medical Center, Leiden, the Netherlands
19 8. Laboratory of Clinical Chemistry and Hematology, Jeroen Bosch Hospital, 's Hertogenbosch,
20 the Netherlands
21 9. Laboratory of Clinical Chemistry and Laboratory Medicine, Dicoon BV, location Canisius
22 Wilhelmina Hospital, Nijmegen, the Netherlands
23 * Equal contributions 24
25 **ORC~~rc~~ID iD**
26 Margaretha G.M Roemer	0000-0003-0220-3496
27 Wendy P.J. den Elzen	0000-0002-6774-4077
28 Rosalinde K.E. Poortvliet	0000-0001-6521-8770
29 Jacobijn Gussekloo	0000-0001-7186-8278
30 Steef Kurstjens	0009-0008-4409-5079
31 Bauke A. de Boer	0000-0002-4572-1157
32 Anne Margreet de Jong	0000-0002-9638-9115 33
34 **Corresponding author**
35 Dr. Anne Margreet de Jong
36 Atalmedial Diagnostics Centre, Amsterdam, The Netherlands
37 Louwesweg 6
38 P.O. Box 69641
39 1066 EC Amsterdam
40 The Netherlands
41 Email: [adejong@atalmedial.nl](mailto:adejong@atalmedial.nl)

42

43 **Declarations**
44 **Conflict of interest:** The authors declare no competing interests.
45 **Funding:** This study had no external funding source.
46 **Ethical approval:** Not applicable.
47 **Author’s contributions:** Conceptualization, study design and data collection: M.G.M.R, W.P.J.d.E.,
48 B.A.d.B. and A.M.d.J.; data preparation and methodology: M.G.M.R, W.P.J.d.E. and A.M.d.J.;
49 investigation: M.G.M.R, W.P.J.d.E. and A.M.d.J.; writing - original draft preparation: M.G.M.R,
50 W.P.J.d.E. and A.M.d.J; writing - review and editing: M.G.M.R, W.P.J.d.E. and A.M.d.J, R.K.E.P., J.G.,
51 S.K., B.A.d.B. All authors have read and agreed to the published version of the manuscript.
52 **Acknowledgements:** not applicable. 53

54	Number of words: 2499 words

55

56	Short title: diagnostic algorithms on anaemia 57

58	Keywords: general practice, anaemia, iron deficiency, ferritin, vitamin B12 deficiency, folate deficiency,

59	guidelines, laboratory aspects

1	**Abstract**

2

3 *Background*
4 Anaemia is highly prevalent and is commonly caused by nutritional deficiencies. Guidelines often
5 include an algorithm to find the~~is~~ underlying cause. Here, we compared diagnostic algorithms and
6 suggested laboratory tests for anaemia in clinical practice guidelines in countries with similar healthcare
7 systems, focusing on iron, vitamin B12 and folate deficiency anaemia in the general adult population. 8
9 *Methods*
10 We searched for guidelines on anaemia in the Trip, Guidelines International Network and country
11 specific databases. To be selected for this systematic review, the guidelines had to include diagnostic
12 criteria or algorithms to determine the cause of anaemia in the general adult population. 13
14 *Results*
15 In total, 14 records were included. For iron deficiency anaemia, guidelines varied in diagnostic criteria
16 ranging from ~~only~~ ferritin only, to ~~up to~~ ferritin in various combinations with a variety of other parameters,
17 with different cut-off values. For vitamin B12 or folate deficiency, besides measurement of vitamin B12
18 or folate, some guidelines mentioned methylmalonic acid or homocysteine. Quality of evidence
19 underlying cut-offs and parameters was variable, and laboratory aspects were underrepresented. 20
21 *Conclusions*
22 There was a lot of variation in the included diagnostic algorithms, especially for iron deficiency anaemia.
23 Differences in cut-off values were seen, even when using similar diagnostic strategies. Furthermore,
24 supporting evidence was variable. Further research is needed to determine optimal algorithms. Our
25 results highlight the need for inclusion of relevant laboratory aspects in guidelines, ~~considering~~
26 appropriate diagnostics and clinical decision limits. 27
1 **Introduction**

2

3 Anaemia is a highly prevalent disorder worldwide affecting up to 25~~4.8~~% of the population [1, 2].

**Field Code Changed**

4 Anaemia is defined as a low number of red blood cells or a low haemoglobin concentration within the
5 red blood cells for the age and sex of the individual [1]. Low haemoglobin concentrations result in a

**Field Code Changed**

6 decreased capacity of the blood to carry oxygen to the tissues in the body. Consequently, symptoms
7 include fatigue, weakness, dizziness and shortness of breath. Anaemia is not a diagnosis in itself, but
8 often a presentation of an underlying condition. Anaemia is most frequently caused by nutrient
9 deficiencies due to inadequate diets or inadequate absorption of nutrients, predominantly iron, vitamin
10 B12 and folate. In addition, chronic diseases, infection, kidney failure, and also genetic disorders, bone
11 marrow pathologies, and acute or chronic haemorrhage can cause anaemia [3, 4]. It is therefore

**Field Code Changed**

12 relevant to diagnose the cause of anaemia to adequately treat the patient.
13 General practitioners play a crucial role in the diagnosis and management of anaemia. The
14 diagnosis~~Analysis~~ of anaemia typically involves a complete blood count (CBC) and further investigative
15 assays to pinpoint the specific aetiology. Premature or unnecessary testing can lead to excessive use
16 of diagnostic blood tests, while failure to test anaemic patients can result in incomplete or missed
17 diagnoses or under- orf overtreatment. Both scenarios can potentially increase healthcare costs [5-7].

**Field Code Changed**

18 To ensure effective and standardised care, anaemia guidelines have been developed. These
19 guidelines typically encompass initial assessment, diagnostic testing, management and treatment,
20 follow-up and monitoring. ~~Adhering to established clinical guidelines is advised to ensure effective and~~
21 ~~standardised care. Given the different~~ For different physiological settings, including~~needs varying by~~
22 age, sex, ~~ethnicity,~~and pregnancy status or for ~~within~~ specific patient groups, ~~a plethora of~~ different
23 guidelines for diagnosing~~analysis of~~ anaemia have been developed. Also, different countries have
24 different requirements for the way in which guidelines are developed, who is involved in their
25 development, and what sources are used. Therefore, the aim of this systematic review was to compare
26 the diagnostic algorithms and suggested laboratory tests for anaemia in clinical practice guidelines in
27 countries with similar healthcare systems for the most common causes of anaemia: iron, vitamin B12
28 and folate deficiency in adults from the primary care population. This may provide guidance for the
29 development of novel guidelines. 30

31

32

33

34

1 **Methods**

2

3 *Data sources and searches*
4 First, we searched for guidelines on anaemia in two guideline databases: Trip database
5 ([https://www.tripdatabase.com](https://www.tripdatabase.com/)) and Guidelines International Network (G-I-N, [http://www.g-i-n.net](http://www.g-i-n.net/)) on

**Field Code Changed**

**Field Code Changed**

6	January 12^th^ 2022. These databases were selected to search for evidence-based guidelines across the

7 world. We performed an advanced search for ‘anemia’ or ‘anaemia’ in both databases. Second, we
8 performed searches in country specific guideline databases (ao. Germany, UK, USA, Canada,
9 Australia) on October 15^th^ 2023. The most recent version of the guidelines was sought and used on

10	February 29th 2024.

11

12 *Guideline selection*
13 To be selected for this systematic review, the guidelines had to include diagnostic criteria or algorithms
14 to find the cause of anaemia in the primary care adult population. We focused on nutritional deficiencies
15 (iron, vitamin B12 and folate). Guidelines had to include criteria for adults; specific guidelines for children
16 were excluded. When multiple guidelines from the same society were found, the most recent version
17 was used. Guidelines focusing on specific populations (e.g. anaemia in pregnancy, chronic kidney
18 disease, dialysis patients, heart failure, cancer), guidelines of which full versions were not available in
19 English, Dutch or German and guidelines of which full versions were inaccessible online or withdrawn,
20 were excluded. Two authors (combinations of MR, WdE, AdJ) independently assessed the titles and
21 full texts of the guidelines retrieved during the searches against the inclusion and exclusion criteria, and
22 selected or rejected as appropriate. Disagreement was resolved by consensus. 23
24 *Data extraction, quality assessment and analysis*
25 The recommendations in the selected guidelines were extracted by two authors (WdE, AdJ) and
26 checked by a third (MR). We rated the quality of the guidelines with a scoring system, based on the
27 evidence underpinning the recommendations. Level 0: no evidence provided, level 1: based on expert
28 opinion, level 2: based on randomised controlled trials / peer reviewed studies before 2010 (for the test
29 of interest), level 3: based on randomised controlled trials / peer reviewed studies 2010 and later (for
30 the test of interest), level 4: based on a systematic review / meta-analysis. Note that guidelines that
31 referred to studies before, as well as after 2010 were evaluated as level 3. 32
33 **Results**
34 A total of 87 records were identified by the combined searches (Supplementary Table 1). Forty-four
35 records were excluded due to the focus on a specific patient group. The remaining 43 full texts were
36 screened and another 29 were excluded in line with the above mentioned exclusion criteria (Figure 1).
37 The 14 included guidelines originated from 6 different countries (US, UK, Germany, Canada, Qatar, the
38 Netherlands).

39

40 *Patient population*
41 We focused on guidelines in the primary care patient population. Nonetheless, a plethora of different
42 anaemia guidelines were found that included specific organs, patient groups and underlying diseases.
1 Therefore, we decided to select all guidelines focusing on the “general population”. This included
2 primary care, as well as secondary care guidelines in otherwise healthy people.
3 Age definitions for patient populations that were included in the different guidelines were
4 variable. Most of the guidelines did not state a specific age definition [8-15], while others focused on

**Field Code Changed**

**Field Code Changed**

**Field Code Changed**

**Field Code Changed**

**Field Code Changed**

**Field Code Changed**

5 age ≥5 years [16], >12 years [17], ≥16 years [18, 19], ≥18 years [20] or on older adults [21]. Although
6 all guidelines acknowledged that older people have a higher risk of developing anaemia, and/or
7 suggested further diagnostics, no age-specific diagnostic algorithms were provided. 8
9 *Definition of anaemia*
10 According to the World Health Organization (WHO), anaemia is defined as haemoglobin levels <12.0
11 g/dL in women and <13.0 g/dL in men [1]. Most of the guidelines adhered to this definition [8, 9, 11-14,

**Field Code Changed**

**Field Code Changed**

**Field Code Changed**

**Field Code Changed**

**Field Code Changed**

12 17, 19, 20], although one guideline used <13.5 g/dL in men [16] and one guideline used <11.5 g/dL in
13 women (Table 1) [18]. Three guidelines did not specify a value for low haemoglobin, but referred to
14 local laboratory reference values [10, 15, 21].

15

16 **Table 1. Summarized haemoglobin cut-offs in the included guidelines to define anaemia**

| **Guideline** | **Year** | **Country** | **Haemoglobin**  **level** | **Evidence** | **Ref** |
| --- | --- | --- | --- | --- | --- |
| NHS Greater Glasgow and Clyde | 2022 | UK | M <13 g/dL,  F <11.5 g/dL | 1 | 18 |
| Centre for Perioperative Care | 2022 | UK | M <13 g/dL,  F <12 g/dL * | 4 | 8 |
| Association of Scientific Medical Societies Germany | 2021 | Germany | M <13 g/dL,  F <12 g/dL | 4 | 9 |
| British Society of Gastroenterology | 2021 | UK | < N | NA | 10 |
| National Institute for Health and Care Excellence | 2021 | UK | M <13 g/dL,  F <12 g/dL | 4 | 17 |
| Dutch College of General Practitioners | 2021 | The  Netherlands | M < 13 g/dL,  F <12 g/dL # | 4 | 11 |
| Ministry of Public Health Qatar | 2020 | Qatar | M <13 g/dL,  F <12 g/dL | 4 | 12 |
| American Gastroenterological Association | 2020 | USA | M <13 g/dL,  F <12 g/dL | 4 | 13 |
| National Institute for Health and Care Excellence | 2020 | UK | M <13 g/dL, F <12 g/dL # | 4 | 19 |
| Royal College of Nursing | 2019 | UK | M <13 g/dL,  F <12 g/dL | 4 | 14 |
| BC guidelines | 2019 | Canada | < N | NA | 15 |
| Association of Scientific Medical Societies Germany | 2018 | Germany | M <13 g/dL,  F <12 g/dL, Drop > 2 g/dL | 4 | 20 |
| Toward Optimized Practice | 2018 | Canada | M <13.5 g/dL,  F <12 g/dL # | 1 | 16 |
| American Medical Directors Association | 2007 | USA | < N | NA | 21 |

17 Abbreviations: Ref, reference; M, male; F, female; N, normal; NA, not applicable
18 * international consensus statement in the peri-operative period: Hb <13 g/dL for both sexes [22]
19 # Suggested cut-off values for assessment

20

21

1 *Iron deficiency anaemia*
2 For iron deficiency anaemia, there was a lot of variation in the included diagnostic criteria, as
3 summarised in Table 2. Diagnostic criteria ranged from only ferritin, up to ferritin in various combinations
4 with mean corpuscular volume (MCV), iron, transferrin, transferrin saturation, total iron binding capacity
5 (TIBC), C-reactive protein (CRP) or erythrocyte sedimentation rate (ESR), and reticulocyte
6 haemoglobin content (Ret-Hb) or reticulocytes, with different cut-off values. Of note, even when a similar
7 strategy was used, differences in cut-off values were seen.
8 Testing of MCV was recommended in seven guidelines, in combination with ferritin [9-12, 16,

**Field Code Changed**

9 20, 21]. Other erythrocyte indices (mean corpuscular haemoglobin, mean corpuscular haemoglobin
10 concentration, red cell distribution width) were variably mentioned in a minority of the guidelines, and
11 therefore not included in Table 2.
12 All guidelines included ferritin for the diagnosis of iron deficiency anaemia. Cut-offs ranged from
13 <15 µg/L [15, 18], <30 µg/L [8, 14, 17, 20] to <45 µg/L [13] or referred to the local lower limit of reference

**Field Code Changed**

**Field Code Changed**

**Field Code Changed**

**Field Code Changed**

**Field Code Changed**

**Field Code Changed**

**Field Code Changed**

**Field Code Changed**

**Field Code Changed**

**Field Code Changed**

**Field Code Changed**

14 range [10-12, 21]. Two guidelines used different ferritin cut-offs for men and women [9, 16].
15 The fact that ferritin is an acute phase protein and thus increases with infection or inflammation
16 was recognized in all guidelines. Nonetheless, some guidelines only mentioned it [9, 12, 13, 17, 21],
17 while other guidelines specified the ferritin level for which iron deficiency could still be considered [11,
18 15, 16, 18, 20] and/or recommended measurement of CRP/ESR to confirm the presence of
19 inflammation [8, 10, 14]. Additional testing of transferrin saturation when a normal ferritin level was
20 found was recommended in five guidelines [8, 10, 14, 20, 21].
21 Testing of iron itself was recommended in three guidelines, in combination with MCV, ferritin,
22 transferrin, transferrin saturation and/or TIBC [11, 12, 21]. Lastly, three guidelines recommended
23 examination of Ret-Hb or reticulocytes [8-10]. 24
25 *Vitamin B12 or folate deficiency anaemia*
26 Six vitamin B12 and folate deficiency anaemia guidelines were included. Most of the guidelines
27 contained a combined anaemia diagnostic algorithm with iron deficiency [8, 11, 12, 20, 21] and one

**Field Code Changed**

**Field Code Changed**

28 guideline specifically focused on vitamin B12 and folate deficiency (Table 3) [19]. All guidelines
29 mentioned that vitamin B12 and/or folate anaemia is macrocytic. Four guidelines stated that the
30 anaemia may also be normocytic [8, 11, 19, 21].

**Field Code Changed**

**Field Code Changed**

31 For vitamin B12, one guideline recommended a specific cut-off [19], the other guidelines
32 referred to local laboratory reference values. With regards to the use of total vitamin B12 (cobalamin)
33 or active vitamin B12 (holotranscobalamin), four guidelines specifically recommended the (initial) testing
34 of total vitamin B12 [8, 11, 19, 20].

**Field Code Changed**

35 In addition to vitamin B12, four guidelines mentioned testing for methylmalonic acid (MMA)
36 and/or homocysteine, always in addition to vitamin B12 [12, 21] or when the vitamin B12 level is in the

**Field Code Changed**

**Field Code Changed**

**Field Code Changed**

**Field Code Changed**

**Field Code Changed**

37 lower normal reference range [11, 20].
38 For folate, most guidelines recommended testing for folate only [8, 11, 21]. Two guidelines
39 included RBC folate as a diagnostic criterion [19, 20]. One guideline recommended lactate
40 dehydrogenase (LDH) and reticulocytes for folate, as well as vitamin B12 deficiency [11].
1 **Table 2. Summarized diagnostic algorithms of the included iron deficiency anaemia guidelines.**

| **Guideline** | **Year** | **Country** | **MCV**  **(fl)** | **Ferritin (µg/L)** | **Iron** | **Transferrin** | **Transferrin sat (%)** | **TIBC** | **CRP (mg/L) or ESR** | **Ret-He / Reticulocytes** | **Evidence** | **Ref** |
| --- | --- | --- | --- | --- | --- | --- | --- | --- | --- | --- | --- | --- |
| NHS Greater Glasgow and Clyde | 2022 | UK |  | < 15 or 15 - 50 † |  |  |  |  |  |  | 1 | 18 |
| Centre for Perioperative Care | 2022 | UK |  | < 30 * or 30 - 100 † |  |  | <20% * |  | > 5 † | < 30 pg * | 3 | 8 |
| Association of Scientific Medical Societies Germany | 2021 | Germany | < 78 | M < 35, F < 23  or ≥ N † |  |  |  |  |  | < 28 pg / ≤ N | 2 | 9 |
| British Society of Gastroenterology | 2021 | UK | < N* | < N or N * |  |  | Low * |  | > N * | < N * | 4 | 10 |
| National Institute for Health and Care Excellence | 2021 | UK |  | < 30 ‡ |  |  |  |  |  |  | 3 | 17 |
| Dutch College of General Practitioners | 2021 | the Netherlands | ≤ N | < N  or < 100 † * | < N * | > N * |  |  |  |  | 2 | 11 |
| Ministry of Public Health Qatar | 2020 | Qatar | < N | ≤ N ‡ | < N |  |  | ≥ N |  |  | 3 | 12 |
| American Gastroenterological Association | 2020 | USA |  | < 45 ‡ |  |  |  |  |  |  | 4 | 13 |
| Royal College of Nursing | 2019 | UK |  | < 30 or 30 - 100 ^ |  |  | < 20% ^ |  | > 5 ^ |  | 1 | 14 |
| BC guidelines | 2019 | Canada |  | < 15 or 15 - 30 $  or < 100 † |  |  |  |  |  |  | 2 | 15 |
| Association of Scientific Medical Societies Germany | 2018 | Germany | < N | < 30 * or 30 - 100 * or > 300 †# |  |  | < 20% * or  †# |  |  |  | 3 | 20 |
| Toward Optimized Practice | 2018 | Canada | < 75 * | M < 30 *, F < 13  or < 100 † |  |  |  |  |  |  | 3 | 16 |
| American Medical Directors Association | 2007 | USA | < N | < N or < N #  or N † | < N # † | > N # | < N # † | > N # |  |  | 2 | 21 |

2 Abbreviations: MCV, mean corpuscular volume; sat, saturation; TIBC, total iron binding capacity; CRP, C-reactive protein; ESR, erythrocyte sedimentation rate; Ret-He, reticulocyte haemoglobin
3 equivalent; Ref, reference; M, male; F, female; N, normal; pg, picograms.
4 * and/or
5 # and
6 ^ or
7 † Patients with chronic (inflammatory) disease, active inflammation or malignancy
8 ‡ Ferritin is an acute phase protein and increases in patients with infection or inflammation
9 $ Probably iron deficiency
1 **Table 3. Summarized diagnostic algorithms of the included vitamin B12 / folate deficiency anaemia guidelines.**

|  | | | | **Vitamin B12** | | | | **Folate** | | | | **Folate and vit. B12** | |  | |
| --- | --- | --- | --- | --- | --- | --- | --- | --- | --- | --- | --- | --- | --- | --- | --- |
| **Guideline** | **Year** | **Country** | **MCV**  **(fl)** | **Vit. B12**  **(pmol/L)** | **Active vit.**  **B12** | **MMA** | **Hcy** | **Folate (nmol/L)** | **RBC**  **folate (nmol/L)** | **MMA** | **Hcy** | **LDH** | **Reticulocytes** | **Evidence** | **Ref** |
| Centre for Perioperative Care | 2022 | UK | ≥ N | < N |  |  |  | < N |  |  |  |  |  | 3 | 8 |
| Dutch College of General Practitioners | 2021 | the Netherlands | ≥ N | < N or low N * |  | Optional (1st) * | Optional (2nd) * | < N |  |  |  | > N | < N | 3 | 11 |
| National Institute for Health and Care Excellence | 2020 | UK | ≥ N | < 148 |  |  |  | < 7 or N # | < 340 # |  |  |  |  | 3 | 19 |
| Ministry of Public Health Qatar | 2020 | Qatar | > N | < N |  | > N |  | < N |  | N |  |  |  | 3 | 12 |
| Association of Scientific Medical Societies Germany | 2018 | Germany | > N | < N or low N * | Optional * | Optional * | Optional * | < N ^ | Confirm |  | Optional |  |  | 3 | 20 |
| American Medical Directors Association | 2007 | USA | ≥ N | < N |  | > N | > N | < N |  |  |  |  |  | 2 | 21 |

2 Abbreviations: MCV, mean corpuscular volume; Vit, vitamin; MMA, methylmalonic acid; Hcy, homocysteine; RBC, red blood cell; LDH, lactate dehydrogenase; Ref, reference; N, normal
3 * and / or
4 # with normal vit. B12
5 ^ related to food intake, a second fasting blood test is required
  1 *Quality of the evidence*
  2 The quality of the included guidelines was evaluated and ranked for the indicated parameters (colour-
  3 coded in Table 1, 2 and 3). To define anaemia, WHO haemoglobin cut-offs were used in most guidelines
  4 [8, 9, 11-14, 17, 19-21]. Given the systematic nature of the evidence and recently unchanged cut-offs

**Field Code Changed**

**Field Code Changed**

**Field Code Changed**

**Field Code Changed**

**Field Code Changed**

**Field Code Changed**

**Field Code Changed**

**Field Code Changed**

5 [23], this was regarded as level 4. For iron deficiency anaemia, two guidelines were considered level 4
6 [10, 13], five guidelines level 3 [8, 12, 16, 17, 20], four guidelines level 2 [9, 11, 15, 21] and two
7 guidelines level 1 [14, 18]. It must be noted that guidelines frequently referred to each other, but
8 nonetheless used variable cut-offs. Five out of six vitamin B12 and folate deficiency anaemia guidelines
9 were considered level 3 [8, 11, 12, 19, 20] and one guideline level 2 [21].

10

11 *Laboratory aspects*
12 Laboratory aspects were only scarcely reported in the included guidelines. Even though laboratory
13 measurements are used in all guidelines, none of the guidelines specified which manufacturer or
14 measurement method was used to determine the specific cut-off values, and the type of material (e.g.
15 plasma or serum) that should be used for the test of interest. 16

17

1 **Discussion**
2 In primary care, the CBC is one of the most common blood tests, often done as a routine check-up.
3 When a low haemoglobin is found, follow-up tests are used to investigate the cause of anaemia [24].

**Field Code Changed**

4 This systematic review elucidated similarities and differences in diagnostic algorithms in evidence-
5 based guidelines for the most common causes of anaemia: iron, vitamin B12 and folate deficiency. An
6 overview of our observations and recommendations for future guidelines are summarised in Figure 2.
7 In brief, we observed that primary and secondary care guidelines are intertwined, and that there is
8 insufficient attention to diversity aspects. Furthermore, a lot of variation was seen in the recommended
9 diagnostic criteria, ~~and~~ cut-off values and evidence underpinning the algorithms, even in the presence
10 of similar diagnostic strategies. Importantly, even though laboratory testing is proposed in all guidelines,
11 the specifics of these tests are not described. 12
13 *Patient population and diversity in diagnostic algorithms*
14 Primary care, as well as secondary care guidelines in otherwise healthy people were included. For
15 future guidelines, a focus on either the primary or secondary care setting is endorsed, as well as
16 alignment between the recommendations of primary care and secondary care. Similar misalignments
17 were observed in the field of subclinical hyperthyroidism [25].

**Field Code Changed**

18 Remarkably, no distinctions were made by sex or age in the majority of the diagnostic
19 algorithms. However, research shows that sex differences may be of importance [26]. Nonetheless, the

**Field Code Changed**

**Field Code Changed**

20 WHO did not find enough evidence for a change of ferritin cut-off values by sex [27]. Additionally, the
21 diagnostic work-up and treatment of anaemia in older patients may be different from other age groups
22 [28-30]. Hence, we recommend including ~~different aspects of diversity,~~ at least age and sex, in the

**Field Code Changed**

23 diagnostic algorithms.

24

25 *Included parameters in diagnostic algorithms*
26 After anaemia is identified, the majority of the guidelines used MCV to define the cause of the anaemia.
27 Nonetheless, a normal MCV does not exclude the presence of iron, vitamin B12 or folate deficiency
28 [31]. Therefore, MCV should be mainly used as a supporting parameter.

**Field Code Changed**

29 For iron deficiency anaemia, ferritin was included in all algorithms. However, the used cut-offs
30 were variable, as has been previously described [32-35] and no proper justification for these cut-offs

**Field Code Changed**

31 was provided. Despite an extensive review performed by the WHO, insufficient evidence was found for
32 a consensus on the ferritin threshold and therefore the cut-off of <15 µg/L remained unchanged [27, 32,

**Field Code Changed**

33 33]. In the presence of inflammation, WHO recommends a cut-off of 70 µg/L to diagnose iron deficiency
34 anaemia [27]. However, most of the guidelines stated a ferritin cut-off of 100 µg/L in this situation. There

**Field Code Changed**

35 is no consensus about which additional parameters should be evaluated. Furthermore, ferritin levels
36 are affected by recent oral iron supplementation intake, coffee and other dietary factors [36]. Therefore,

**Formatted:** English (United States)

37 it is recommended to fast and withhold supplements before measuring ferritin [37]. Nonetheless, these
38 factors are not emphasized in guidelines, and therefore this can lead to diagnostic confusion.
39 For vitamin B12 and folate deficiency, most guidelines referred to local lower limit of reference
40 values. Given the variation of used cut-offs in literature [38, 39], this is a sensible approach. Not all

**Field Code Changed**

1 guidelines specify if total vitamin B12 or active vitamin B12 should be tested. Also, additional testing
2 with MMA and homocysteine is not consistently advised. To date, the most reliable parameter to detect
3 vitamin B12 deficiency is still a matter of debate. According to Herbert’s hypothesis, active vitamin B12
4 is an earlier and more sensitive indicator of vitamin B12 deficiency [40, 41], which was supported by

**Field Code Changed**

5 later studies [42-45]. Nonetheless, other studies contradict this and state that reliability of active and

**Field Code Changed**

6 total vitamin B12 is similar [46, 47].

**Field Code Changed**

7 These findings highlight the need for additional research to determine the most optimal
8 parameters and cut-offs to diagnose anaemia caused by nutritional deficiencies. 9
  10 *Laboratory aspects in guidelines*
  11 There is limited awareness of laboratory aspects in anaemia guidelines, while all the algorithms use
  12 laboratory tests to determine the cause of anaemia. It must be noted that the ferritin, vitamin B12 and
  13 folate assays are not harmonised between platforms and there are substantial inter-analyser
  14 differences. The lack of harmonisation may result in different values when the same~~a~~ sample is
  15 measured in different labs using different analyser platforms [48-52]. Concentrations may also be

**Field Code Changed**

16 dependent on the type of material, e.g. serum or plasma that is used for the analysis. In general, serum
17 as well as plasma is acceptable for analysis. Nonetheless, higher or lower values may be found in
18 specific types of material for ferritin, vitamin B12 and folate [44, 51, 53]. Therefore, if cut-offs are

**Field Code Changed**

19 mentioned, the used sample type, analyser platform and method should be specified. Applying
20 inappropriate cut-offs lead to misinterpretation and may cause under- or overdiagnosis of patients.
21 Given the lack of harmonisation of ferritin, vitamin B12 and folate, at this point we recommend referring
22 to local laboratory reference values or cut-offs provided by the local laboratory. 23

24 *Strengths and limitations*
25 To our knowledge, this is the first study that systematically reviewed anaemia guidelines focusing on
26 the diagnostics of the most common causes of anaemia. In addition to summarising diagnostic
27 strategies, we present recommendations that may be used for developing new guidelines. A limitation
28 of this study is that we only selected guidelines of countries with comparable healthcare systems that
29 were available in Dutch, German or English, potentially missing other relevant guidelines. Nonetheless,
30 our review provides an overview of diagnostic strategies from guidelines from all over the world. 31
32 *Conclusion*
33 All in all, there was a lot of variation in the included diagnostic criteria for anaemia caused by nutritional
34 deficiencies in guidelines for the adult population. Even when similar diagnostic strategies were used,
35 differences in cut-off values were seen and the supporting evidence was variable. Further research is
36 needed to determine optimal algorithms. Even though laboratory tests are a fundamental aspect of the
37 diagnostic algorithms, description of relevant laboratory aspects was lacking. For future guidelines, it
38 will be important to include relevant laboratory aspects in guidelines, considering appropriate
39 diagnostics and clinical decision limits. 40

41

1 **Supporting information**

**Formatted:** Font: Bold, English (United States)

**Formatted:** English (United States)

2

3	Supplementary Table 1. Exclusion table 4

5	Supplementary Table 2. PRISMA abstract checklist 6

**Formatted:** English (United States)

7	Supplementary Table 3. PRISMA checklist

8

1 **References**
2 1.	World Health Organization. Worldwide prevalence of anaemia 1993-2005 - WHO global
3 database on anaemia. Edited by Bruno de Benoist, Erin McLean, Ines Egli and Mary Cogswell 2008.
4 2.	Safiri S, Kolahi AA, Noori M, Nejadghaderi SA, Karamzad N, Bragazzi NL, et al. Burden of
5 anemia and its underlying causes in 204 countries and territories, 1990-2019: results from the Global
6 Burden of Disease Study 2019. J Hematol Oncol. 2021;14(1):185.
7 3.	Cascio MJ, DeLoughery TG. Anemia: Evaluation and Diagnostic Tests. Med Clin North Am.
8 2017;101(2):263-84.
9 4.	Kassebaum NJ, Collaborators GBDA. The Global Burden of Anemia. Hematol Oncol Clin North
10 Am. 2016;30(2):247-308.
11 5.	de Jong AM, Bozic C, Candido F, van Stralen KJ, de Boer BA. [The cause of anemia is
12 underdiagnosed in primary care]. Ned Tijdschr Geneeskd. 2023;167.
13 6.	Zhi M, Ding EL, Theisen-Toupal J, Whelan J, Arnaout R. The landscape of inappropriate
14 laboratory testing: a 15-year meta-analysis. PLoS One. 2013;8(11):e78962.
15 7.	Kip MMA, Oonk MLJ, Levin MD, Schop A, Bindels PJE, Kusters R, et al. Preventing overuse of
16 laboratory diagnostics: a case study into diagnosing anaemia in Dutch general practice. BMC Med
17 Inform Decis Mak. 2020;20(1):178.
18 8.	Centre for Perioperative Care. Guideline for the Management of Anaemia in the Perioperative
19 Pathway. Available from [https://cpoc.org.uk/guidelines-resources-guidelines/anaemia-perioperative-](https://cpoc.org.uk/guidelines-resources-guidelines/anaemia-perioperative-pathway)
20 [pathway.](https://cpoc.org.uk/guidelines-resources-guidelines/anaemia-perioperative-pathway) 2022.
21 9.	Behnisch W, Muckenthaler M, Kulozik A. Arbeitsgemeinschaft der Wissenschaftlichen
22 Medizinischen	Fachgesellschaften.Eisenmangelanamie.	Available	from
23 [https://register.awmf.org/de/leitlinien/detail/025-021.](https://register.awmf.org/de/leitlinien/detail/025-021) 2021.
24 10.	Snook J, Bhala N, Beales ILP, Cannings D, Kightley C, Logan RPH, et al. British Society of
25 Gastroenterology. Guidelines for the Management of Iron Deficiency Anaemia in Adults. Gut.
26 2021;70:2030-51.
27 11.	Bouma M, Burgers JS, Drost B, Den Elzen WPJ, Luchtman T, Oosterhuis WP, et al. Nederlands
28 Huisarts	Genootschap	(NHG)-Standaard	Anemie	(M76).	Available	from
29 [https://richtlijnen.nhg.org/standaarden/anemie.](https://richtlijnen.nhg.org/standaarden/anemie) 2021.
30 12.	Ministry of Public Health Qatar. National Clinical Guidelines: The Assessment & Management
31 of Common Anaemias in Adults. Available from [https://www.isbtweb.org/resource/the-assessment-and-](https://www.isbtweb.org/resource/the-assessment-and-management-of-common-anaemias-in-adults--1--pdf.html)
32 [management-of-common-anaemias-in-adults--1--pdf.html.](https://www.isbtweb.org/resource/the-assessment-and-management-of-common-anaemias-in-adults--1--pdf.html) 2020.
33 13.	Ko CW, Siddique SM, Patel A, Harris A, Sultan S, Altayar O, et al. American
34 Gastroenterological Association. AGA Clinical Practice Guidelines on the Gastrointestinal Evaluation of
35 Iron Deficiency Anemia. Gastroenterology. 2020;159:1085-94.
36 14.	Royal College of Nursing. Iron deficiency anaemia in adults. Available from
37 [https://www.rcn.org.uk/Professional-Development/publications/pub-007460.](https://www.rcn.org.uk/Professional-Development/publications/pub-007460) 2019.
38 15.	Ministry of Health, British Columbia. Doctors of BC. BCGuidelines. Iron Deficiency - Diagnosis
39 and Management. Available from [https://www2.gov.bc.ca/gov/content/health/practitioner-professional-](https://www2.gov.bc.ca/gov/content/health/practitioner-professional-resources/bc-guidelines/iron-deficiency)
40 [resources/bc-guidelines/iron-deficiency.](https://www2.gov.bc.ca/gov/content/health/practitioner-professional-resources/bc-guidelines/iron-deficiency) 2019.
41 16.	Toward Optimized Practice Iron Defiency Anemia Committee. Iron deficiency anemia clinical
42 practice	guideline.	Edmonton,	AB:	Toward	Optimized	Practice.	Available	from
43 [https://www.albertadoctors.org/media/atabokv2/iron-deficiency-anemia-guideline.pdf.](https://www.albertadoctors.org/media/atabokv2/iron-deficiency-anemia-guideline.pdf) 2018.
44 17.	National Institute for Care and health Excellence (NICE). Iron deficiency anaemia. Available
45 from cks.nice.org.uk 2021.
46 18.	NHS Greater Glasgow and Clyde. Clinical guideline. Iron deficiency Anaemia in Adults: Oral
47 and	Intravenous	Iron	Therapy	Treatment.	Available	from
48 [https://rightdecisions.scot.nhs.uk/m/2295/ida.pdf.](https://rightdecisions.scot.nhs.uk/m/2295/ida.pdf) 2022.
49 19.	National Institute for Care and health Excellence (NICE). Anaemia - B12 and folate deficiency.
50 Available from cks.nice.org.uk. 2020.
51 20.	Deutsche Gesellschaft fur Anasthesiologie und Intensivmedizin in Kooperation mit der
52 Arbeitsgemeinschaft der Wissenschaftlichen Medizinischen Fachgesellschaften. Praoperative Anamie:
53 Diagnostik	und	Therapie	der	Praoperative	Anamie.	Available	from
54 [https://register.awmf.org/de/leitlinien/detail/001-024.](https://register.awmf.org/de/leitlinien/detail/001-024) 2018.
55 21.	American Medical Directors Association. Anemia in the long-term care setting. Available from
56 [https://paltc.org/products/anemia-long-term-care-setting.](https://paltc.org/products/anemia-long-term-care-setting) 2007.
57 22.	Munoz M, Acheson AG, Auerbach M, Besser M, Habler O, Kehlet H, et al. International
58 consensus statement on the peri-operative management of anaemia and iron deficiency. Anaesthesia.
59 2017;72(2):233-47.
1 23.	World Health Organization. Guideline on haemoglobin cutoffs to define anaemia in individuals
2 and populations. 2024.
3 24.	Fanshawe TR, Ordonez-Mena JM, Turner PJ, Bruel AVD, Shine B, Hayward GN. Frequencies
4 and patterns of laboratory test requests from general practice: a service evaluation to inform point -of-
5 care testing. J Clin Pathol. 2018;71(12):1065-71.
6 25.	Ursem SR, Boelen A, Bruinstroop E, Elders PJM, Gussekloo J, Poortvliet RKE, et al. A
7 systematic review of subclinical hyperthyroidism guidelines: a remarkable range of recommendations.
8 Eur Thyroid J. 2024;13(3).
9 26.	Levi M, Simonetti M, Marconi E, Brignoli O, Cancian M, Masotti A, et al. Gender differences in
10 determinants of iron-deficiency anemia: a population-based study conducted in four European
11 countries. Ann Hematol. 2019;98(7):1573-82.
12 27.	World Health Organization. WHO guideline on use of ferritin concentrations to assess iron
13 status in individuals and populations. 2020.
14 28.	den Elzen WP, Westendorp RG, Frolich M, de Ruijter W, Assendelft WJ, Gussekloo J. Vitamin
15 B12 and folate and the risk of anemia in old age: the Leiden 85-Plus Study. Arch Intern Med.
16 2008;168(20):2238-44.
17 29.	den Elzen WP, Gussekloo J. Anaemia in older persons. Neth J Med. 2011;69(6):260-7.
18 30.	Smelt AF, Gussekloo J, Bermingham LW, Allen E, Dangour AD, Eussen SJ, et al. The effect of
19 vitamin B12 and folic acid supplementation on routine haematological parameters in older people: an
20 individual participant data meta-analysis. Eur J Clin Nutr. 2018;72(6):785-95.
21 31.	Schop A, Stouten K, Riedl JA, van Houten RJ, Leening MJG, Bindels PJE, et al. The accuracy
22 of mean corpuscular volume guided anaemia classification in primary care. Fam Pract. 2021;38(6):735-
23 9.
24 32.	Garcia-Casal MN, Pasricha SR, Martinez RX, Lopez-Perez L, Pena-Rosas JP. Are Current
25 Serum and Plasma Ferritin Cut-offs for Iron Deficiency and Overload Accurate and Reflecting Iron
26 Status? A Systematic Review. Arch Med Res. 2018;49(6):405-17.
27 33.	Garcia-Casal MN, Pasricha SR, Martinez RX, Lopez-Perez L, Pena-Rosas JP. Serum or
28 plasma ferritin concentration as an index of iron deficiency and overload. Cochrane Database Syst Rev.
29 2021;5(5):CD011817.
30 34.	Peyrin-Biroulet L, Williet N, Cacoub P. Guidelines on the diagnosis and treatment of iron
31 deficiency across indications: a systematic review. Am J Clin Nutr. 2015;102(6):1585-94.
32 35.	Richardson D, Brown K, Rusch J, Verburgh E, Louw V, Opie J. From the marrow to the blood:
33 optimising the diagnosis of iron deficiency in the setting of inflammation. Pathology. 2025;57(1):87-93.
34 36.	von Siebenthal HK, Moretti D, Zimmermann MB, Stoffel NU. Effect of dietary factors and time
35 of day on iron absorption from oral iron supplements in iron deficient women. Am J Hematol.
36 2023;98(9):1356-63.
37 37.	Balendran S, Forsyth C. Non-anaemic iron deficiency. Aust Prescr. 2021;44(6):193-6.
38 38.	Raghavan R, Ashour FS, Bailey R. A Review of Cutoffs for Nutritional Biomarkers. Adv Nutr.
39 2016;7(1):112-20.
40 39.	Aparicio-Ugarriza R, Palacios G, Alder M, Gonzalez-Gross M. A review of the cut-off points for
41 the diagnosis of vitamin B12 deficiency in the general population. Clin Chem Lab Med. 2015;53(8):1149-
42 59.
43 40.	Herbert V. The 1986 Herman award lecture. Nutrition science as a continually unfolding story:
44 the folate and vitamin B-12 paradigm. Am J Clin Nutr. 1987;46(3):387-402.
45 41.	Herbert V. Staging vitamin B-12 (cobalamin) status in vegetarians. Am J Clin Nutr. 1994;59(5
46 Suppl):1213S-22S.
47 42.	Heil SG, de Jonge R, de Rotte MC, van Wijnen M, Heiner-Fokkema RM, Kobold AC, et al.
48 Screening for metabolic vitamin B12 deficiency by holotranscobalamin in patients suspected of vitamin
49 B12 deficiency: a multicentre study. Ann Clin Biochem. 2012;49(Pt 2):184-9.
50 43.	Jarquin Campos A, Risch L, Nydegger U, Wiesner J, Vazquez Van Dyck M, Renz H, et al.
51 Diagnostic Accuracy of Holotranscobalamin, Vitamin B12, Methylmalonic Acid, and Homocysteine in
52 Detecting B12 Deficiency in a Large, Mixed Patient Population. Dis Markers. 2020;2020:7468506.
53 44.	Nexo E, Hoffmann-Lucke E. Holotranscobalamin, a marker of vitamin B-12 status: analytical
54 aspects and clinical utility. Am J Clin Nutr. 2011;94(1):359S-65S.
55 45.	Valente E, Scott JM, Ueland PM, Cunningham C, Casey M, Molloy AM. Diagnostic accuracy of
56 holotranscobalamin, methylmalonic acid, serum cobalamin, and other indicators of tissue vitamin
57 B(1)(2) status in the elderly. Clin Chem. 2011;57(6):856-63.
58 46.	Golding PH. Holotranscobalamin (HoloTC, Active-B12) and Herbert's model for the
59 development of vitamin B12 deficiency: a review and alternative hypothesis. Springerplus.
60 2016;5(1):668.
1 47.	Remacha AF, Sarda MP, Canals C, Queralto JM, Zapico E, Remacha J, et al. Role of serum
2 holotranscobalamin (holoTC) in the diagnosis of patients with low serum cobalamin. Comparison with
3 methylmalonic acid and homocysteine. Ann Hematol. 2014;93(4):565-9.
4 48.	Hoofnagle AN. Harmonization of blood-based indicators of iron status: making the hard work
5 matter. Am J Clin Nutr. 2017;106(Suppl 6):1615S-9S.
6 49.	Devalia V, Hamilton MS, Molloy AM, British Committee for Standards in H. Guidelines for the
7 diagnosis and treatment of cobalamin and folate disorders. Br J Haematol. 2014;166(4):496-513.
8 50.	Choy KW, Sezgin G, Wijeratne N, Calleja J, Liwayan R, Rathnayake G, et al. Assessment of
9 analytical bias in ferritin assays and impact on functional reference limits. Pathology. 2022;54(3):302-
10 7.
11 51.	Owen WE, Roberts WL. Comparison of five automated serum and whole blood folate assays.
12 Am J Clin Pathol. 2003;120(1):121-6.
13 52.	Kurstjens S, van Dam AD, Oortwijn E, den Elzen WPJ, Candido F, Kusters R, et al.
14 Inconsistency in ferritin reference intervals across laboratories: a major concern for clinical decision
15 making. Clin Chem Lab Med. 2025;63(3):600-10.
16 53.	Ji Y, Faddy H, Hyland C, Flower R. A plasma ferritin is not always a serum ferritin. Pathology.
17 2015;47:S89-S90.

18

Response to Reviewers

Dear dr. Mayne,

Thank you for the evaluation of our manuscript: **Diagnostic algorithms in guidelines on anaemia: a systematic review**. We appreciate your and the reviewers’ careful assessment and the thoughtful comments regarding the work. We carefully evaluated each point and believe that addressing these comments helped us to improve the quality of the manuscript.

All modifications have been highlighted in the marked-up copy of our manuscript. Below please find a point-by-point response to your and the reviewers’ comments and journal requirements.

Sincerely,

Anne Margreet de Jong Laboratory specialist

#### Comments of academic editor

The reviewers suggested some minor amendments including some grammatical changes and in addition a few references to support the ferritin analysis. I also feel that the proposed title change (review of nutritional anaemias) is a good suggestion.

Thank you for the evaluation of our manuscript. We agree with the minor amendments and proposed title change and have adjusted the manuscript accordingly.

#### Review comments to the Author

Reviewer #1: Thank you for the opportunity to review your manuscript that investigates similarities and differences in diagnostic algorithms of clinical treatment guidelines of nutritional anaemia in adult patients.

Major observations:

1. While many of the PRISMA 2020 checklist items were followed, I consider this manuscript to represent a narrative and thematic synthesis describing and comparing the guidelines and their recommendations. The quality of evidence and strength of recommendations contained in each clinical practice guideline were not evaluated using a recognised approach e.g., a rapid (iCAHE) or a complex instrument (the GRADE, AGREE II and AGREE-REX). In this respect I consider the methodology to fall short of a complete systematic review. I question the application of the scoring system used in the manuscript to rate the quality of the guidelines (p.5 3rd paragraph, 2^nd^ sentence) as a suitable alternative. Table 1 shows three CPGs (8[2022], 14[2019], and 20[2018]) with similar diagnostic algorithms for iron deficiency yet variable evidence.

We acknowledge the lack of a recognized grading approach for the different guidelines. However, it is important to note that we did not evaluate guidelines as a whole, but specifically focused on the laboratory parameters within the guidelines and the evidence that was used to support the different diagnostic algorithms. Consequently, the same guideline may have a different quality for the different nutritional anaemias, e.g. iron deficiency anaemia (evidence = 2) and vitamin B12/folate deficiency anaemia (evidence = 3) in the Dutch anaemia guideline.

Similarly, even though a similar diagnostic algorithm is used in different guidelines, the evidence supporting this algorithm may differ. To clarify this, we changed the following sentence in the discussion on page 10, line 10: “Furthermore, a lot of variation was seen in the recommended diagnostic criteria, cut-off values and evidence underpinning the algorithms, even in the presence of similar diagnostic strategies.”

We do agree that the wording in our title: “systematic review” may be misleading. Therefore, we removed this from the title and changed it to: “Comparison of diagnostic algorithms in guidelines on nutritional anaemias in adults”.

Minor observations:

1. I believe the title of the manuscript should be amended to reflect the focus of the work: e.g., Diagnostic algorithms in guidelines on nutritional anaemias in adults.

Thank you, we agree with this suggestion and have adjusted the title to: “Comparison of diagnostic algorithms in guidelines on nutritional anaemias in adults”.

2. While Qatar’s health care system is a leader in the Gulf Cooperation Council, is it really similar to the healthcare systems of e.g., Western European countries in this study? Developing vs. developed health care system? As an example, in 2020 Qatar’s health care spending has exceeded its European counterparts but it was predominantly toward secondary care (70-75%), with ~20-25% for primary care and <5% for self-care. The comparative breakdown for the Netherlands was ~45%, ~45%, and ~10% for secondary, primary, and self- care, respectively.

There is certainly a difference between health care systems in different countries, but this is true even within Western European countries. For instance: in the Dutch health care system, the general practitioner acts as a gatekeeper for referral to medical specialists, while in the German health care system, patients have direct access to medical specialists. Qatar has consistently ranked among the top healthcare systems globally and we therefore believe that it is justified to include the Qatar anaemia guideline in this manuscript.

3. p.4. 2nd paragraph 2nd last sentence: amend under- of overtreatment to under- or overtreatment

Done.

4. p.5 3rd paragraph 1st and 3rd sentence: repetitive: To ensure effective and standardised care, anaemia guidelines have been developed. Adhering to established clinical guidelines is advised to ensure effective and standardised care. Suggest consolidation.

Thank you for pointing this out. We removed the second sentence.

5. p.5 3rd paragraph 4th sentence: I am unsure how physiological needs vary by ethnicity. Reconsider or substantiate.

Agree. We reworded the sentence to: “For different physiological settings, including age, sex and pregnancy status or for specific patient groups, different guidelines for diagnosing anaemia have been developed.”

6. References should be extensively reviewed to comply with the journal’s author guideline. In particular; 1. Use the Vancouver referencing style and, for consistency, restrict authors to the first six followed by et al. and 2. Review referencing style of internet resources. Some of the listed URLs are stale.

Done, see also point 6 of journal requirements.

7. Abbreviations used in the tables should be explained in the caption e.g., 8. The ferritin cut- off of reference 9 in Table 2 appears erroneous.

We added a caption explaining the abbreviations to the tables.

Reviewer #2: This is a highly relevant and useful article which is well-written.

Some minor grammatical edits are required, and additional suggested references are provided to include important pre-analytical factors affecting serum ferritin levels which are not widely recognized and can lead to diagnostic confusion.

We would like to thank the reviewer for her/his appreciative comments and for the effort invested in evaluating our work. We incorporated the suggested edits in the attached document. See specifics below:

Edits in introduction:

- grammar: to find the underlying cause
- grammar: from ferritin only, to ferritin in various combinations...
- suggest reword: affecting up to 25%...
- add: red blood cells for the age and sex of the individual.
- reword: The diagnosis of anaemia typically includes ...
- suggest reword: physiological settings including age, sex, ... different guidelines for the diagnosis of anaemia have been developed.

Edits in discussion:

- the CBC
- suggest delete.

reword: we recommend including at least age and sex in the diagnostic algorithms

- when the same sample is measured

Edits in Table 1:

- The (capitalise)
- This international statement Hb specifically refers to the pre-operative management of anaemia. Please add this into the legend:

* International consensus statement for pre-operative anaemia: Hb< 13 g/dl for both sexes.

Edits in Figure 2:

- use MCV as a supportive parameter only

Furthermore, we incorporated the suggested references in the text.

Specifically, on page 10, we added the reference to the ferritin cut-off study to the following sentence on line 30: “For iron deficiency anaemia, ferritin was included in all algorithms. However, the used cut-offs were variable, as has been previously described”

In addition, we added the following sentence to the discussion, page 10, lines 35-38: “Furthermore, ferritin levels are affected by recent oral iron supplementation intake, coffee and other dietary factors. Therefore, it is recommended to fast and withhold supplements before measuring ferritin. Nonetheless, these factors are not emphasized in guidelines, and therefore this can lead to diagnostic confusion.”

#### Journal requirements

When submitting your revision, we need you to address these additional requirements.

1. Please ensure that your manuscript meets PLOS ONE's style requirements, including those for file naming.

Done.

2. We note that your Data Availability Statement is currently as follows: [All relevant data are within the manuscript and its Supporting Information files.]

Please confirm at this time whether or not your submission contains all raw data required to replicate the results of your study.

Submission contains all data required.

If there are ethical or legal restrictions on sharing a de-identified data set, please explain them in detail (e.g., data contain potentially sensitive information, data are owned by a third-party organization, etc.) and who has imposed them (e.g., an ethics committee). Please also provide contact information for a data access committee, ethics committee, or other institutional body to which data requests may be sent. If data are owned by a third party, please indicate how others may request data access.

There are no ethical or legal restrictions.

3. Please include a copy of Table 1, 2 & 3 which you refer to in your text on page 7.

Tables are placed in the manuscript file directly after the paragraph in which it is first cited, as described in the journal requirements.

4. Please include captions for your Supporting Information files at the end of your manuscript, and update any in-text citations to match accordingly. Please see our Supporting Information guidelines for more information: [http://journals.plos.org/plosone/s/supporting-information.](http://journals.plos.org/plosone/s/supporting-information)

Supporting Information files are updated.

#### Supporting information

Supplementary Table 1. Exclusion table Supplementary Table 2. PRISMA abstract checklist Supplementary Table 3. PRISMA checklist

5. As required by our policy on Data Availability, please ensure your manuscript or supplementary information includes the following:

A numbered table of all studies identified in the literature search, including those that were excluded from the analyses. For every excluded study, the table should list the reason(s) for exclusion.

If any of the included studies are unpublished, include a link (URL) to the primary source or detailed information about how the content can be accessed.

A table of all data extracted from the primary research sources for the systematic review and/or meta-analysis. The table must include the following information for each study:

Name of data extractors and date of data extraction

Confirmation that the study was eligible to be included in the review.

All data extracted from each study for the reported systematic review and/or meta-analysis that would be needed to replicate your analyses.

If data or supporting information were obtained from another source (e.g. correspondence with the author of the original research article), please provide the source of data and dates on which the data/information were obtained by your research group.

We generated a table containing all identified guidelines in our database search. The dates of the search and date of assessment and consensus was added. Furthermore, we added the reason for exclusion. See Supplementary Table 1.

If applicable for your analysis, a table showing the completed risk of bias and quality/certainty assessments for each study or outcome. Please ensure this is provided for each domain or parameter assessed. For example, if you used the Cochrane risk-of-bias tool for randomized trials, provide answers to each of the signalling questions for each study. If you used GRADE to assess certainty of evidence, provide judgements about each of the quality of evidence factor. This should be provided for each outcome.

Not applicable

An explanation of how missing data were handled. Not applicable

This information can be included in the main text, supplementary information, or relevant data repository. Please note that providing these underlying data is a requirement for publication in this journal, and if these data are not provided your manuscript might be rejected.

6. Please review your reference list to ensure that it is complete and correct. If you have cited papers that have been retracted, please include the rationale for doing so in the manuscript text, or remove these references and replace them with relevant current references. Any changes to the reference list should be mentioned in the rebuttal letter that accompanies your

revised manuscript. If you need to cite a retracted article, indicate the article’s retracted status in the References list and also include a citation and full reference for the retraction notice.

Done, reference list was updated.
