# Supplementary material for: Comparison of diagnostic algorithms used in guidelines on nutritional anaemias in adults
Source: PLoS One. 2025 Oct 3;20(10):e0333688. doi: 10.1371/journal.pone.0333688 (PMC12494270; doi:10.1371/journal.pone.0333688)
Supplement: Attachment — Submitted filename: Response to reviewers.docx [file pone.0333688.s008.docx]

6. Review Comments to the Author

Please use the space provided to explain your answers to the questions above. You may also include additional comments for the author, including concerns about dual publication, research ethics, or publication ethics. (Please upload your review as an attachment if it exceeds 20,000 characters)

Reviewer #1: All my comments have been adequately addressed. I have no more concerns on the content of the manuscript.

Thank you for the evaluation of our revised manuscript.

Reviewer #2: I have further reviewed and provided detailed comments and edits on the manuscript in track changes.
Whilst improved, the manuscript still requires further minor revisions and another round of review.

Thank you for the evaluation of our revised manuscript.

We agree with most of the suggestions, which have been adjusted (for details see the manuscript with track changes). In brief, we changed the title, Figure 2 and most of the grammatical suggestions were incorporated. In addition we removed the term ‘systematic review’ in the whole manuscript, and clarified the terminology on reticulocyte counts and CRP/ESR. In addition the Footnotes for Tabel 2 and 3 are adjusted.
